# Peripheral and central inflammation in progressive supranuclear palsy syndrome: an immunological and imaging neuroinflammation study

**DOI:** 10.3389/fimmu.2026.1841265

**Published:** 2026-07-23

**Authors:** Saikat Dey, Aishwarya Kumar, Pardeep Kumar, Paranthaman V. Kavya, Sandipan Mondal, Vikram V. Holla, Nitish Kamble, Rohan Mahale, Pramod K. Pal, Ravi Yadav, Monojit Debnath

**Affiliations:** 1 Department of Human Genetics, National Institute of Mental Health and Neurosciences, Bangalore, India; 2 Department of Neuroimaging & Interventional Radiology, National Institute of Mental Health and Neurosciences, Bangalore, India; 3 Department of Neurology, National Institute of Mental Health and Neurosciences, Bangalore, India

**Keywords:** inflammasome, neuroimaging, neuroinflammation, progressive supranuclear palsy, systemic inflammation, Th17 pathway

## Abstract

**Introduction:**

Neuroinflammation plays an important role in the pathobiology of Progressive Supranuclear Palsy Syndrome (PSP-S). However, it is not adequately known whether peripheral inflammation correlates to neuroinflammation in PSP-S. This study aimed to examine a link between peripheral and brain inflammation in PSP-S by integrating blood and cerebrospinal fluid (CSF) inflammatory profile and Positron Emission Tomography (PET)-Magnetic Resonance Imaging (MRI) (PET-MRI).

**Methods:**

Fifty-six PSP-S patients and equal number of healthy controls were recruited. Plasma Th17 pathway cytokine (IL-6, IL-1β, IL-4, IL-10, IL-17A, IL-17F, IL-21, IL-22, IL-23, IL-25, IL-31, IL-33, IFN-γ, sCD40L, and TNF-α) levels were measured in all the study participants. CSF levels of these cytokines were measured only in PSP-S patients. The expressions of NF-κB (*Nfkb1,* and *Nfkb2)*, inflammasome (*Nlrp3, Casp1*, and *Il18)*, Th17 (*Il1b, Il6, Il17, Tnfa, Il22, Il23, Rorc,* and *Stat3)* and anti-inflammatory (*Tgfb,* and *Il10*) genes were quantified in all the study participants. PET-MRI was carried out in a subset of PSP-S patients (n=12) and eleven disease controls (Parkinson’s Disease=7, and Multiple System Atrophy=4).

**Results:**

Inflammasome (*Casp1* and *Il18*), NF-κB (*Nfkb1)* and inflammatory (*Il1b* and *Il6*) genes were upregulated in PSP-S patients. Plasma IL-1β, IL-6, IL-17A, and IL-17F levels were significantly elevated in PSP-S patients. IL-1β levels significantly correlated between plasma and CSF. PET-MRI indicated neuroinflammation in several brain regions of PSP-S patients.

**Conclusion:**

Both systemic inflammation and neuroinflammation are evident in PSP-S. The positive correlation of higher plasma IL-1β levels with neuroinflammation provides preliminary evidence towards the influence of specific peripheral inflammatory molecule on neuroinflammatory basis of PSP-S. The altered immune elements of the current study further reinforce the pathogenic relevance of immune-inflammatory pathways in PSP-S pathobiology.

## 1 Introduction

Progressive Supranuclear Palsy Syndrome (PSP-S) is a rapidly progressive neurodegenerative disorder with clinical manifestations of gait disturbance, vertical gaze palsy, recurrent falls, akinesia, and cognitive dysfunction (1). Pathologically, PSP-S is defined by the accumulation of hyperphosphorylated tau protein leading to neuronal loss in the basal ganglia, brainstem, cerebellum and, to a lesser extent, the cerebral cortex, which is consistent across the autopsy studies (2, 3). The heterogeneous nature of the disease, as well as variability of progression and predominant symptomatology, has led to the classification of several clinical variants of PSP-S (1, 4, 5). Despite the advances in genetic, epigenetic, and neuropathological understanding, the precise mechanistic basis of PSP-S remains inadequately understood.

Emerging studies suggest a pivotal role of neuroinflammation in toxic tau accumulation and the progression of tauopathies (6–8). Microglia activation and subsequent neuroinflammation, oxidative stress, neurotoxicity, and neurodegeneration have been implicated in PSP-S and other tauopathies (9, 10). Evidence of neuroinflammation also emerged from studies reporting significantly elevated levels of pro-inflammatory cytokines (TNF-α, IL-1β, and IL-6) in the cerebrospinal fluid (CSF) and upregulated *Il1b* transcript levels in the substantia nigra of PSP-S brains (11, 12). Besides neuroinflammation, a few recent studies have also indicated altered peripheral immune cells and molecules in PSP-S (13, 14). A higher neutrophil-to-lymphocyte ratio (NLR), a general indicator of peripheral inflammation, was reported in PSP-S (13, 15). The differences in the plasma and CSF levels of IL-1β and IL-6 were reported between PSP-P and PSP-RS and such distinct inflammatory patterns were suggested to contribute to various PSP-S phenotypes and clinical course (16). It is noteworthy that the peripheral immune markers were shown to be associated with greater clinical severity, higher expression of neurodegeneration markers, and more pronounced region-specific brain atrophy in PSP-S (14). In an interesting study, elevated peripheral immune parameters such as IL-1β, NLR, and platelet-to-lymphocyte ratio (PLR) were associated with lower Montreal Cognitive Assessment (MoCA) test score, implying an impact of peripheral inflammation on cognitive decline in PSP-S (17). In a recent genome-wide association study (GWAS), a novel susceptibility locus was identified at complement factor 4A (C4A) and C4A was found to co-localize with abnormal tau species in oligodendrocytes (18). These findings indicate involvement of innate immunity in PSP-S pathobiology. It is important to note that several factors and mechanisms such as genetic background, metabolic pathways, neurotrophic factors, and glial cells are crucially involved in the regulation of immune and inflammatory responses. A pivotal role of genetic regulation of immune and inflammatory responses is also becoming increasingly apparent in PSP-S (19). Glial activation, altered expression of neurotrophic factors like glial cell-derived neurotrophic factor (GDNF) and an impact of inflammatory as well as neurotrophic factors on quality of life in patients with PSP-S were reported in recent studies (18, 20, 21). Increased leukocyte metabolism leading to its metabolic reprogramming and activation of NRF2/HO-1 pathway, a master regulator of inflammatory and metabolic response was demonstrated in PSP-S (15). Despite these advances, a question remained pertinent and unanswered for decades is whether systemic inflammation contributes to neuroinflammatory changes in neurodegenerative diseases. In an interesting recent study, peripheral inflammation was shown to be associated with neuroinflammation and worse clinical outcome in frontotemporal lobar degeneration (22). On the contrary, a neuroimaging study conducted on a small cohort has raised doubt about unequivocal role of non-specific inflammatory markers in neurodegenerative changes in PSP-S (23). Overall, a peripheral to central inflammation continuum model seems to operate in tauopathies. However, this proposition has not yet been empirically examined in PSP-S. Further, the currently available data on immune dysregulations in PSP-S are discrete in nature and do not inform the involvement of any specific immune pathway(s).

The inflammatory sequelae are mediated by a cascade of immune events, including activation of nuclear factor-κB (NF-κB), inflammasome, and inflammatory T lymphocytes. To delineate the immunopathogenesis of PSP-S, it is important to examine the implications of the crucial inducers, regulators (positive and negative) and effectors of inflammation. Inflammasomes are the most potent inducers of inflammation and are implicated in the neuroinflammatory and neurodegenerative diseases (24). NF-κB is a family of inducible transcription factors and comprises five structurally related members (NF-κB1, NF-κB2, RelA, RelB, and c-Rel). NF-κB regulates the expression and function of a large number of genes involved in immune and inflammatory responses (25). NF-κB is crucial in regulating the activation of inflammasome (26). Besides this, NF-κB is also involved in the regulation of activation, differentiation and effector functions of inflammatory T cells. T helper 17 (Th17) is an inflammatory lineage of T lymphocytes. NF-κB activation leads to Th17 cell differentiation through induction of IL-1, IL-6, and IL-23 as well as regulation of master regulator transcription factors of Th17 cells (27). Noncanonical NF-κB promotes the pathological attributes of Th17 cells in mediating neuroinflammation (28). Notably, inflammasome also activates Th17 cells (29). The above understanding suggests potential interactions amongst NF-κB, inflammasome, and Th17 cells in driving the inflammatory responses. Currently, there is a lack of studies examining synergistic effects of these elements of the immune system in PSP-S. To address this knowledge gap, this study aimed to decipher the role of NF-κB, inflammasome and Th17 cells in elucidating a mechanistic connection between peripheral and central inflammation in PSP-S. An integrated study design was adopted to measure levels of a panel of fifteen Th17 pathway-related cytokines in the plasma and CSF, quantify expression of NF-κB, inflammasome and Th17 pathway-related genes in the lymphocytes and perform imaging neuroinflammation in patients with PSP-S.

## 2 Study participants and methods

In this cross-sectional case-control study, fifty-six patients with PSP-S were recruited from the in-patient and out-patient services of the Movement Disorders Unit of the Department of Neurology, National Institute of Mental Health and Neurosciences (NIMHANS), Bangalore, India, from January 2022 to December 2025. The patients were recruited as per the criteria developed by the Movement Disorder Society (MDS)-2017 (1). Furthermore, the mandatory inclusion criteria included sporadic occurrence, age more than 40 years at the onset of the first PSP-S-related symptom and gradual progression of PSP-S-related symptoms. Clinical and demographic details, including age at onset, sex, addiction history, cognitive impairments, and disease severity, were documented. Cognitive decline was assessed using the MoCA test score, while disease severity was evaluated using the Progressive Supranuclear Palsy Rating Scale (PSPRS) and Part III of the Unified Parkinson’s Disease Rating Scale (UPDRS-III). Additionally, 56 age- and gender-matched healthy controls with no neurological, psychological, and immune disorders were recruited from the community. A subset of PSP-S patients (n=12) was considered for Positron Emission Tomography (PET)-Magnetic Resonance Imaging (MRI) (PET-MRI). Additionally, eleven disease controls (Parkinson’s Disease, PD = 7 and Multiple System Atrophy-P, MSA-P=4) were used as a reference group to compare the magnitude of neuroinflammation between PSP-S patients and disease controls. The ethical approval was obtained from the Institutional Ethics Committee (No: NIMHANS/DO/IEC (BS & NS)/2022 dated 17^th^ January 2022) of NIMHANS. Written informed consent was obtained from each participant prior to their recruitment into the study.

### 2.1 Collection of the biological samples and processing

Approximately 10 ml of whole blood sample was collected by venepuncture of the median cubital vein into EDTA tubes under aseptic conditions. Approximately 3 ml of whole blood was utilized for genomic DNA extraction, while the remaining 7 ml was processed for the isolation of plasma and peripheral blood mononuclear cells (PBMCs). Whole blood was first centrifuged at 4000 rpm for 10 minutes at 4°C, and PBMCs were subsequently isolated by density gradient centrifugation using Ficoll-Paque PLUS 1.077. Additionally, 2 ml of CSF was collected from a subset of patients (n=28) by lumbar puncture and centrifuged at 4000 rpm for 10 minutes at 4°C to remove any cell debris and red blood cells. All samples were aliquoted and stored at -80°C until analysis.

### 2.2 Estimation of plasma and CSF levels of the cytokines

Plasma levels of 15 cytokines were measured using Bio-Plex Pro™ Human Th17 Cytokine Panel 15-Plex on a Bio-Plex 200 platform (Bio-Rad, Hercules, CA, USA) in all the study participants. However, the CSF levels of these cytokines were measured only in patients with PSP-S to correlate the cytokine profile between plasma and CSF of patients with PSP-S. To compare the CSF levels of cytokines between PSP-S and healthy controls, the data of CSF levels of IL-1β, IL-6, IL-10, IL-17A, IL-17F, IL-21, IL-22 and IL-23 in healthy controls (n=37) were taken from our earlier study (30). The analytes of the Th17 panel were estimated in pg/ml, and the limit of detection (LOD) of each cytokine was as follows: IL-6 (0.52 pg/ml), IL-1β (0.02 pg/ml), IL-4 (0.67 pg/ml), IL-10 (0.30 pg/ml), IL-17A (0.49 pg/ml), IL-17F (0.80 pg/ml), IL-21 (2.13 pg/ml), IL-22 (0.30 pg/ml), IL-23 (1.55 pg/ml), IL-25 (0.07 pg/ml), IL-31 (0.58 pg/ml), IL-33 (0.58 pg/ml), IFN-γ (0.43 pg/ml), sCD40L (0.41 pg/ml), and TNF-α (0.07 pg/ml). All the samples were run in duplicates. A standard curve was generated for each analyte prior to every run. The data were analyzed using Bioplex Manager software (version 6.1).

### 2.3 RNA extraction and quantification of gene expression

Total RNA was isolated from PBMCs using a commercially available QIAamp RNA Blood Mini-kit (Qiagen, Limburg, The Netherlands) following the manufacturer’s instructions. The RNA was converted to complementary DNA (cDNA) using a cDNA Reverse Transcription Kit (Takara Bio, USA). Fifteen genes representing NF-κB (*Nfkb1*, and *Nfkb2)*, inflammasome (*Nlrp3, Casp1*, and *Il18)*, Th17 pathway (*Il1b, Il6, Il17, Tnfa, Il22, Il23, Rorc*, and *Stat3)* and anti-inflammatory pathway (*Tgfb*, and *Il10*) were considered for quantification of gene expression based on their functional relevance. The TaqMan probes of all the genes were obtained from Applied Biosystems (CA, USA). The assay IDs of the genes are shown in **Supplementary Table 1**. The expression levels of *Il1b*, *Il6*, *Il17*, *Rorc*, *Stat3*, *Tgfb*, *Tnfa*, and *Il22* genes were quantified in all the participants using TaqMan Gene Expression Assays on a Quant Studio 6 real-time PCR system (Applied Biosystems, CA, USA). The reaction mix was prepared by adding TaqMan Universal PCR Master Mix (Takara Bio, USA), TaqMan probes, cDNA template and 6-carboxyl-X-Rhodamine dye (ROX) in the reaction tube. The amplifications of all the genes were normalized using TATA-box binding protein (*Tbp*) as an internal control. Additionally, each sample was run in triplicate with a no-template control (NTC), and a calibrator in each plate to neutralize inter-assay variability. The final data were analyzed by the 2^-ΔΔCt^ method. The expression levels of *Casp1*, *Nlrp3*, *Il10*, *Il18*, *Il23a*, *Nfkb1*, and *Nfkb2* genes were measured by droplet digital PCR in a QX200 AutoDG Droplet Digital PCR System (Bio-Rad, USA) using TaqMan Probes. The reactions were prepared by adding TaqMan probes with ddPCR™ Supermix for Probes (No dUTP) (Bio-Rad, USA) with cDNA templates. Approximately 20,000 droplets were generated from each sample and the PCR was performed in a thermocycler (Bio-Rad, USA). The assay was performed with an NTC in a 96-well format with an internal control gene (*Tbp*) according to the manufacturer’s instructions.

### 2.4 PET-MRI and data analysis for assessing microglial activation

The radioactive ligand [^11^C]-PBR28 was used to assess microglial activation through translocator protein (TSPO) PET-MRI scanning. The binding affinity of [^11^C]-PBR28 is known to be influenced by a single-nucleotide polymorphism (SNP), rs6971 of the TSPO gene (31). Genotyping of rs6971 was performed for the patients prior to initiating the PET-MRI.

#### 2.4.1 Genomic DNA extraction and genotyping

The genomic DNA was extracted from the whole blood using QIAamp DNA Blood Mini Kit (Qiagen, Limburg, The Netherlands). The genotyping of rs6971 (Assay ID: C_2512465_20) was performed using TaqMan biallelic discrimination assay on a Quant Studio real-time 6 PCR system (Applied Biosystems, CA, USA). The reaction mix contained TaqMan Universal PCR Master Mix (Takara Bio, USA), TaqMan probe, ROX and template genomic DNA. The assay was performed with an NTC.

#### 2.4.2 PET-MRI data acquisition and analysis

Patients consenting to PET imaging underwent simultaneous PET-MRI on SIEMENS Biograph mMR (Erlangen, Germany). Approximately 110 MBq of [^11^C]-PBR28 ligand was injected intravenously and flushed with 20 ml normal saline prior to the scanning procedure. PET images were acquired using the following parameters: 500 mm Field of view (FOV), 400 mm anterior-posterior FOV, 1.0 zoom, 3 interactions, 21 subsets, HD PET reconstruction method, and 2.0 mm Gaussian filter. Besides, the MRI images were acquired simultaneously using sequences such as multiple gradient echo three-dimensional magnetization-prepared rapid acquisition (MPRAGE) images [echo time TE = 2.33; repletion time TR = 2200; flip angle = 8 degrees; field of view FOV = 250; slice orientation= PSL; 1mm isotropic voxels] and diffusion images [b value = 1000 s/mm2, TE = 90; TR = 3900; diffusion directions - 6; FOV- 230; slice orientation= LAS; 4.0 mm isotropic voxels].

PET-MR images were processed using SYNGO Via (VB30) and subsequently analyzed with the PNEURO module in PMOD 4.4. Standardized uptake values (SUVs) were measured in the twenty different functionally relevant brain regions, such as, dorsal striatum, lentiform nucleus, precentral gyrus, hippocampus, amygdala, parahippocampal area, postcentral gyrus, cuneus, caudate nucleus, nucleus accumbens, putamen, thalamus, pallidum, substantia nigra, insula, white matter (WM), grey matter, cerebellum WM, brainstem, and whole brain. The SUVs were then compared to the cerebellum grey matter to obtain a final SUV-R score, which was further used for the analysis. Additionally, volumes of these regions were calculated from MRI scans.

### 2.5 Statistical analyses

All the statistical analyses were performed using SPSS (Version 25) and Jamovi (Version 2.6.44). The normality of data was checked using the Kolmogorov-Smirnov (KS) test. For normally distributed data, equality of variances was tested using Levene’s test, followed by Student’s t-test to compare the two groups. For the data having a non-normal distribution, Mann-Whitney U test was used to compare the variables between the groups and Spearman’s rank correlation for obtaining the correlation coefficients between variables. False discovery rate (FDR) adjusted *p*-values were calculated using the Benjamini-Hochberg method and provided alongside the raw *p*-values. The graphical representations were plotted in GraphPad Prism (Version 8.0) and RStudio (4.5.1). A *p*-value of <0.05 was considered significant.

## 3 Results

### 3.1 Clinical and demographic data of the study participants

The mean age of the PSP-S cohort was 60.61 ± 7.24 years, with a modest male dominance (male: female, 34:22), and the mean age of healthy controls was 59.29 ± 8.55 years. The PSP-S cohort was further subdivided into two groups: PSP-Richardson syndrome (PSP-RS) and PSP-nonRS, comprising PSP-Parkinsonism (PSP-P), PSP-progressive gait freezing (PSP-PGF), PSP-frontal (PSP-F), PSP-speech/language (PSP-SL), PSP-corticobasal syndrome (PSP-CBS), PSP-oculomotor (PSP-OM), and PSP-postural instability (PSP-PI). The mean age at onset of the first PSP-related symptom was 58.05 ± 6.99 years, and the mean disease duration was 29.48 ± 15.62 months. A significant difference in disease duration was observed between the PSP-RS and PSP-nonRS groups (*p* = 0.046). The mean MoCA score was 16.64 ± 7.43, while the mean PSPRS and UPDRS-III scores were 38.42 ± 13.15 and 34.54 ± 12.61, respectively. No significant differences were noted in cognitive impairment or motor dysfunction scores between the PSP-RS and PSP-nonRS groups. The PSPRS total score was further subdivided into six components: History (C1), Mentation (C2), Bulbar (C3), Ocular (C4), Limb (C5), and Gait (C6). The clinical and demographic data are summarized in **Table 1**.

**Table 1:** Clinico-demographic data of the study participants.

Variables	Patients (n=56)		Controls (n=56)	Statistics	p value
	PSP-RS(n=29)	PSP-nonRS (n=27)			
Age (years)	60.61 ± 7.24		59.29 ± 8.55	1.421*	0.158
Sex (Male: Female)	34:22		34:22	–	–
Addiction history (+) (tobacco/alcohol)	10	9	–	0.008†	1.000
Age of onset (years)	57.72 ± 6.80	58.41 ± 7.28	–	-0.363*	0.718
Duration of disease (months)	25.38 ± 10.67	33.89 ± 18.84	–	-2.060*	0.046
MoCA score	15.55 ± 5.65	17.81 ± 8.92	–	-1.124*	0.267
PSPRS total score	38.72 ± 12.57	38.11 ± 13.97	–	0.173*	0.863
PSPRS_C1	9.31 ± 3.93	7.67 ± 4.10	–	1.531*	0.132
PSPRS_C2	2.00 (4.00, 1.00)	1.00 (4.00, 0.00)	–	-0.343‡	0.732
PSPRS_C3	2.00 (3.00, 1.00)	2.00 (3.00, 1.00)	–	-0.192‡	0.895
PSPRS_C4	8.48 ± 3.74	8.81 ± 3.44	–	-0.345*	0.847
PSPRS_C5	4.69 ± 2.0	5.96 ± 3.29	–	-1.735*	0.090
PSPRS_C6	11.86 ± 5.13	11.07 ± 4.54	–	0.607*	0.546
UPDRS-III score	34.59 ± 12.65	34.48 ± 12.81	–	0.031*	0.976

*Independent sample t-test, values are given in mean ± SD; ^†^Chi-square test; ^‡^Mann-Whitney U test, values are given in median (Q3, Q1).

### 3.2 Activation of NF-κB, inflammasome pathways, and peripheral inflammation in PSP-S

**Figure 1:**
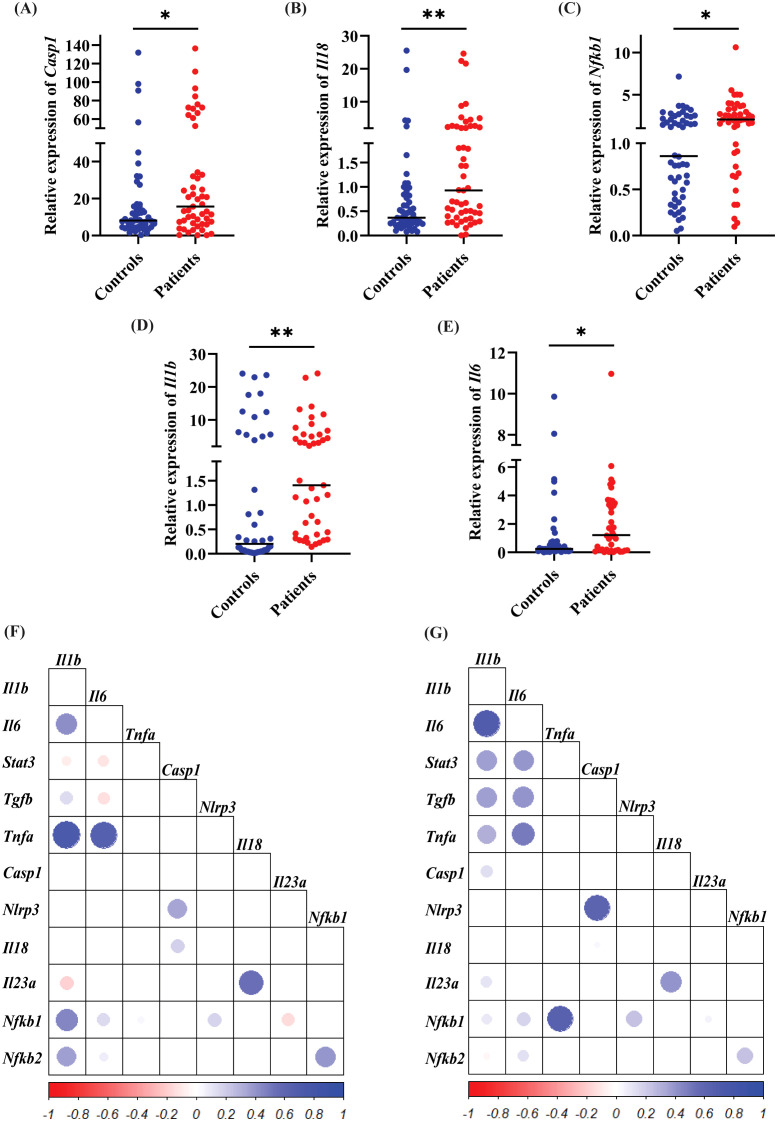
Depicts comparisons of expression of inflammation pathway-related genes between patients with PSP-S and healthy controls. Comparison of expression of **(A)** *Casp1*, **(B)** *Il18*, **(C)** *Nfkb1*, **(D)** *Il1b*, and **(E)** *Il6* genes between patients with PSP-S and healthy controls. Correlogram showing the interactions among the genes of NF-κB, inflammasome and Th17 pathways in **(F)** patients and **(G)** healthy controls. The size of the circle indicates strength, and the color represents the linearity. Significance level *<0.05; **<0.01.

The expressions of inflammasome genes such as *Casp1* (*p* = 0.035) and *Il18* (*p* = 0.001) (**Figures 1A, B**; **Supplementary Table 2**), NF-κB gene, *Nfkb1* (*p* = 0.047) (**Figure 1C** and **Supplementary Table 2**) and inflammatory genes, *Il1b* (*p* = 0.006) and *Il6* (*p* = 0.031) (**Figures 1D, E**; **Supplementary Table 2**) were significantly upregulated in PSP-S patients. However, only *Il1b* and *Il18* remained significant after FDR correction. Additionally, none of the studied genes showed significant differences between PSP-RS and PSP-nonRS subgroups (**Supplementary Table 3**). The correlations between the interacting molecules were also examined (**Figures 1F, G**; **Supplementary Tables 4, 5**). Significant positive correlations between (i) *Il6* and *Tnfa*, and (ii) *Nlrp3*, and *Casp1* genes were observed in both PSP-S and healthy controls. Besides this, some distinct correlation patterns were also found in patients and controls. In the control group, *Il1b* and *Il6* correlated with *Stat3* and *Tgfb*, whereas a moderately strong correlation between *Il1b* and *Tnfa* was observed only in patients.

### 3.3 Activation of inflammatory Th17 pathway in patients with PSP-S

**Figure 2:**
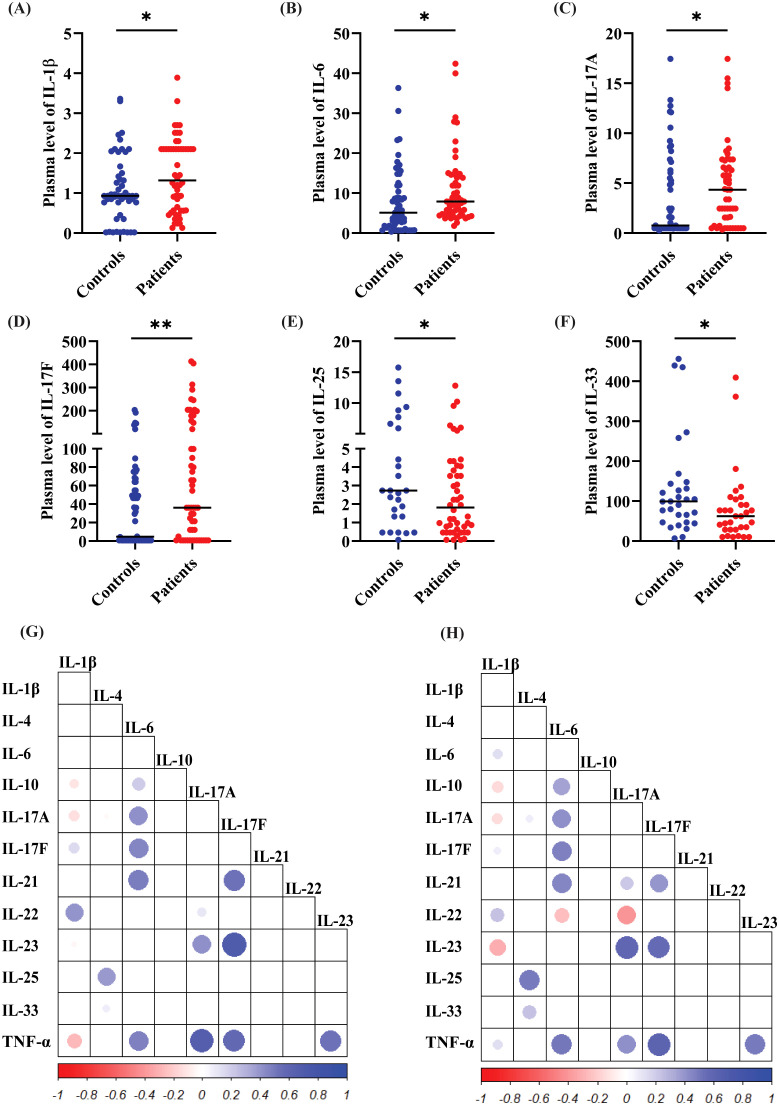
Depicts comparisons of plasma levels of Th17 pathway-related cytokines between patients with PSP-S and healthy controls. Comparison of plasma level of **(A)** IL-1β, **(B)** IL-6, **(C)** IL-17A, **(D)** IL-17F, **(E)** IL-25, and **(F)** IL-33 cytokines between patients with PSP-S and healthy controls. Correlogram showing the interactions among the components of the Th17 pathway in **(G)** patients and **(H)** healthy controls. The size of the circle indicates strength, and the color represents the linearity. Significance level *<0.05; **<0.01.

Significantly elevated IL-1β (*p* = 0.038), IL-6 (*p* = 0.010), IL-17A (*p* = 0.024), and IL-17F (*p* = 0.004) and reduced IL-25 (*p* = 0.039) and IL-33 (*p* = 0.015) levels were observed in the plasma of patients with PSP-S compared to healthy controls (**Figures 2A–F**; **Supplementary Table 6**). However, none of these were significant after FDR correction. The plasma levels of all the studied cytokines were comparable between PSP-RS and PSP-nonRS subgroups (**Supplementary Table 7**). Further, we checked the correlations between the inducers (IL-1β, IL-6), mediator (IL-23) and effector (IL-17A, IL-17F, IL-21, IL-22, and TNF-α) cytokines of Th17 pathway. Plasma IL-1β and IL-6 correlated positively with IL-22 and IL-21, respectively only in patients, while IL-6 showed positive correlations with IL-17A, IL-17F, and TNF-α both in patients and healthy controls. Plasma IL-23 correlated positively with IL-17A, IL-17F and TNF-α in both the patients and controls (**Figures 2G, H**; **Supplementary Tables 8, 9**). Plasma cytokine levels were correlated with their corresponding gene expression levels (**Supplementary Table 10**), however, none of the correlations appeared significant.

### 3.4 Activation of Th17 pathway in CSF and correlation between plasma and CSF levels of cytokines in PSP-S

The CSF levels of IL-1β (p=0.001), IL-6 (p<0.001), IL-17A (p<0.002), IL-22 (p<0.003), and IL-23 (p<0.004) were significantly higher in patients with PSP-S compared to healthy controls (**Table 2**). The levels of all the cytokines were further assessed for correlations between plasma and CSF only in the patient group (**Supplementary Table 11**). Interestingly, a significant positive correlation was observed only for IL-1β (rho=0.602; *p* = 0.004) between plasma and CSF of patients with PSP-S (**Supplementary Figure 1**). However, it showed trend level significance after FDR correction.

**Table 2:** Differences in observed CSF levels of cytokines between the patient and control groups.

Cytokines	PSP-S (n=28)	Controls (n=37)	Statistics*	*p*-value	Adjusted *p*-value
IL-1β	1.140 (1.700, 0.135)	0.150 (0.160, 0.130)	-3.274	0.001	0.001
IL-6	8.625 (11.577, 4.865)	0.410 (0.470, 0.155)	-5.818	<0.001	0.001
IL-17A	10.980 (15.277, 8.617)	0.350 (0.500, 0.250)	-6.864	<0.001	0.001
IL-22	36.035 (102.910, 14.355)	0.330 (0.885, 0.150)	-4.857	<0.001	0.001
IL-23	121.630 (315.550, 79.705)	5.410 (8.000, 4.410)	-5.770	<0.001	0.001

*Mann-Whitney U test.

### 3.5 Correlations of gene expression, plasma, and CSF cytokine profiles with clinical severity

**Figure 3:**
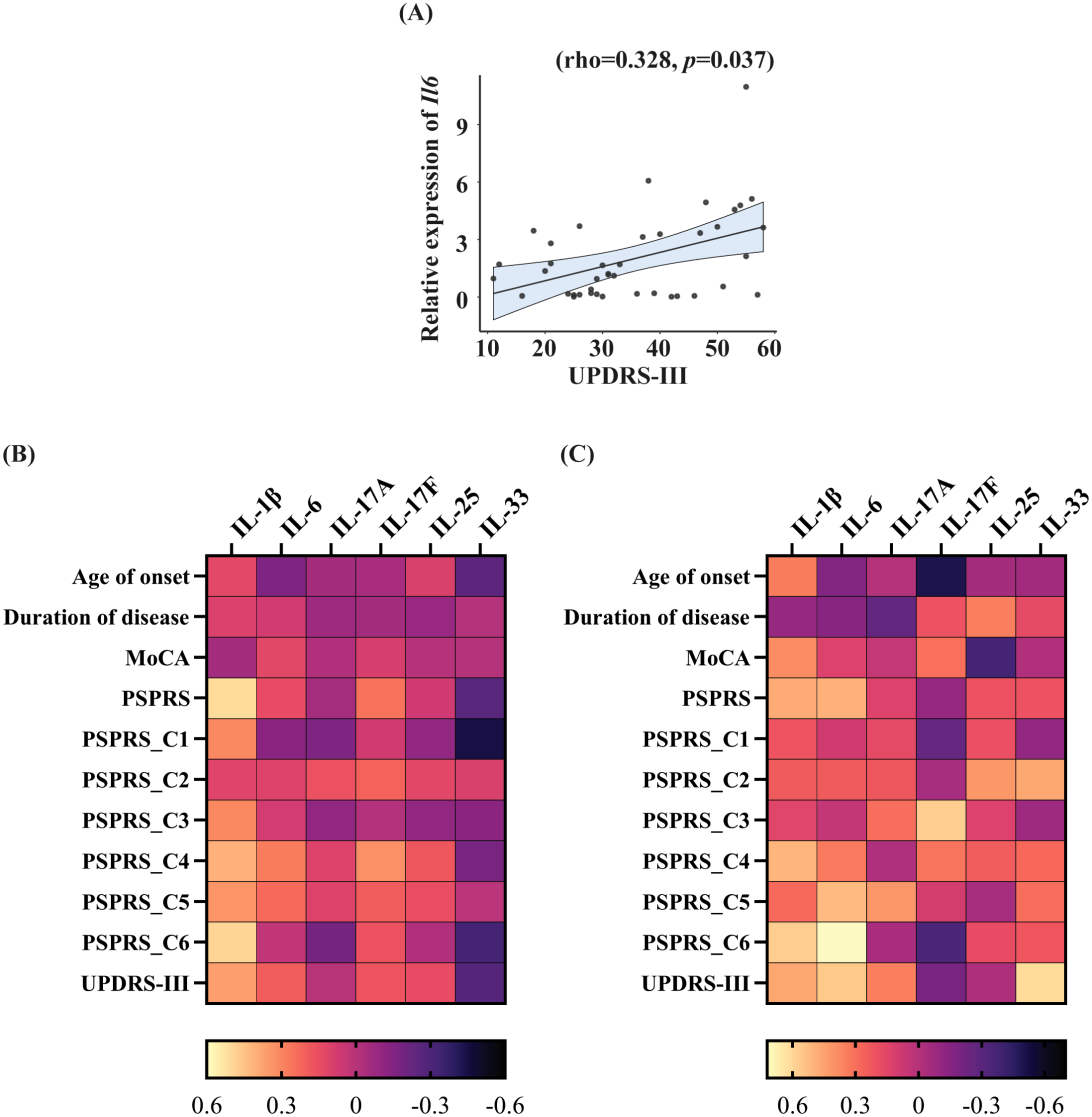
Correlation between plasma, and CSF cytokines with clinical variables. **(A)** Correlation between gene expression of *Il6* and UPDRS-III score. Heatmap showing the correlations of clinical variables with **(B)** plasma and **(C)** CSF levels of cytokines. The color bar represents the linearity of correlation.

All genes, plasma cytokines, and corresponding CSF cytokines were correlated with clinical severity parameters (**Supplementary Table 12-14**). At the transcript level, *Il6* expression positively correlated with the UPDRS-III score (**Figure 3A**). However, the significance was lost after FDR correction. Plasma IL-1β levels showed a positive correlation with the PSPRS total score and its components (C1, C3, C4, C5, and C6), as well as with the UPDRS-III score (**Figure 3B**). Additionally, PSPRS component C4 was associated with plasma levels of IL-6 and IL-17F. On the other hand, a significant negative correlation was observed between plasma IL-33 levels and PSPRS component C1 in patients. Among these, the correlations between plasma IL-1β and PSPRS total score and its components (C4, C6) remained significant after FDR correction. Whereas in CSF, IL-1β sustained its association with PSPRS total score and its components (C4 and C6), as well as UPDRS-III score (**Figure 3C**). Moreover, CSF IL-6 levels showed positive correlations with the PSPRS total score, components C5 and C6, and the UPDRS-III score. A significant positive correlation was also detected between CSF IL-17A levels and PSPRS component C5. However, except for the positive correlation between CSF IL-6 levels and the PSPRS component C6, all other observed correlations lost significance after FDR corrections.

### 3.6 PET findings suggest evidence of neuroinflammation in PSP-S

**Figure 4:**
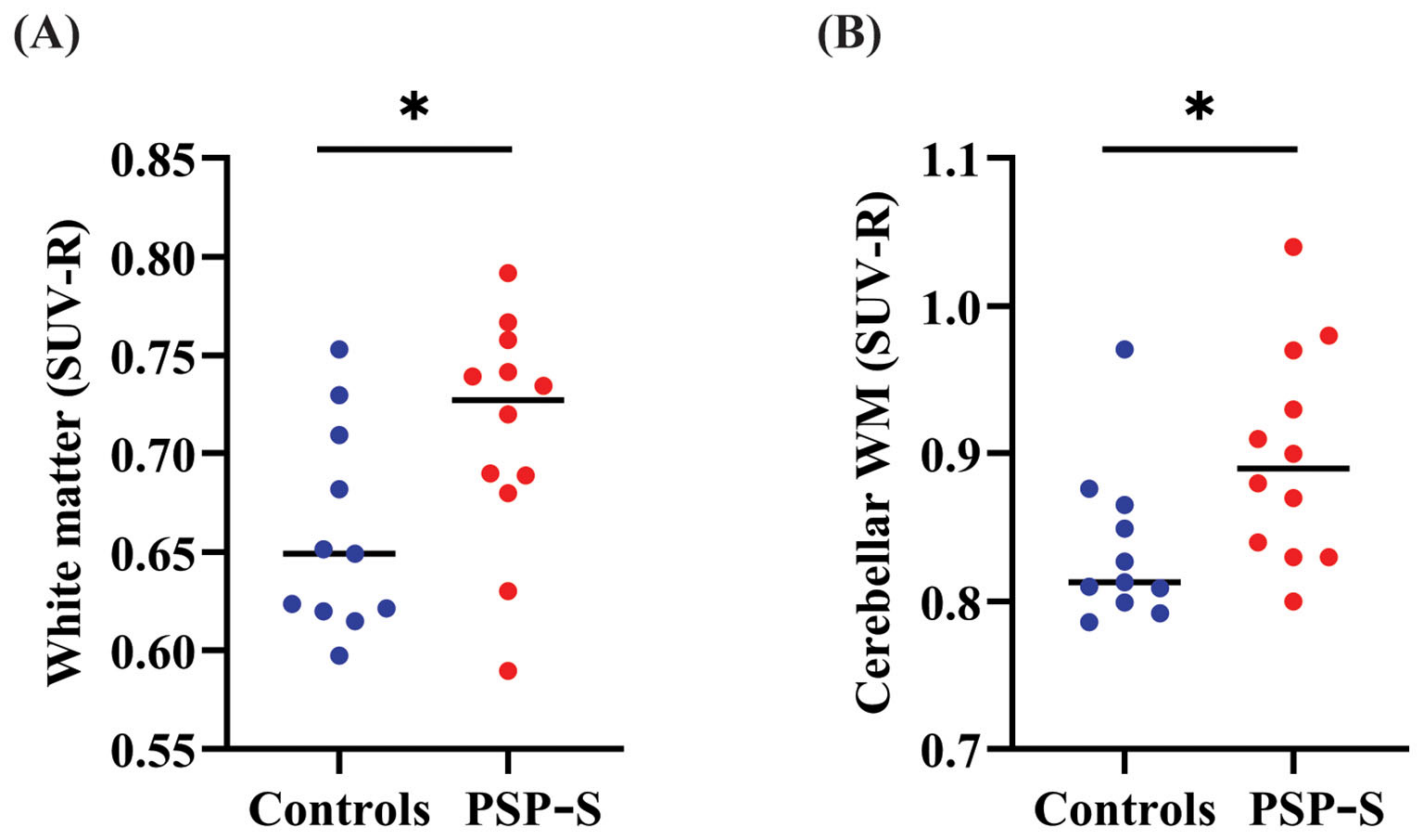
Depicts comparisons of microglia activation between PSP-S and disease controls. Comparison of SUV-R of **(A)** WM and **(B)** cerebellar WM between patients with PSP-S and disease controls. Significance level *<0.05.

Twelve patients were recruited for PET-MRI, all of whom exhibited either high (*n* = 7) or medium (*n* = 5) binding affinity for the PET ligand. Additionally, eleven disease controls (PD = 7 and MSA-P=4) were used as a reference group to compare the magnitude of neuroinflammation between PSP-S patients and disease controls using a one-tailed independent t-test. This specific statistical test was employed to detect the magnitude of neuroinflammation in PSP-S rather than bidirectional differences. Among the disease control group, 8 participants were classified as high-affinity binders and 3 as medium-affinity binders for the PET ligand. Significantly higher inflammation was observed in the WM (*p* = 0.018) and cerebellar WM (*p* = 0.014) regions in PSP-S patients compared to disease controls (**Figures 4A, B**; **Supplementary Table 15**). However, the significance was lost after the FDR correction. Additionally, SUV-R values from twenty brain regions were correlated with the plasma and CSF cytokines as well as clinical profile of the patients with PSP-S (**Supplementary Table 16**). Interestingly, plasma IL-25 level negatively correlated with inflammation in the precentral gyrus region (rho=-0.652, *p* = 0.041), and inflammation of this region positively correlated with the duration of the disease (rho=0.604, *p* = 0.038) (**Supplementary Figures 2A, B**). Plasma IL-25 level was also inversely associated with the inflammation of postcentral gyrus (rho=-0.529, *p* = 0.047), nucleus accumbens (rho=-0.625, *p* = 0.020), and grey matter (rho=-0.662, *p* = 0.013) regions, while plasma IL-33 level inversely correlated with the SUV-R of pallidum region (rho=-0.703, *p* = 0.039) (**Supplementary Figures 2C–F**). On the contrary, CSF level of IL-1β positively correlated with the inflammation in the nucleus accumbens region (rho=1.000, *p* = 0.017) while CSF level of IL-6 positively correlated with the inflammation in the whole brain (rho=1.000, *p* = 0.017), WM (rho=1.000, *p* = 0.017), and cerebellar WM (rho=0.900, *p* = 0.037) in PSP-S patients (**Supplementary Figures 2G–J**). Notably, none of these correlations remained significant after FDR corrections. On the other hand, patients with a higher inflammation in the hippocampus (rho=-0.792, *p* = 0.002), amygdala (rho=-0.697, *p* = 0.012), parahippocampal area (rho=-0.736, *p* = 0.006) and insula regions (rho=-0.588, *p* = 0.044) had an earlier age of onset of first PSP-related symptoms (**Supplementary Table 17**). Among these, only the negative correlation between inflammation in hippocampus and earlier age of onset remained significant after FDR correction. However, no significant correlations were found between SUV-R values and the MoCA score, PSPRS total score, or the UPDRS-III score.

### 3.7 Brain morphometric changes in patients with PSP-S

**Figure 5:**
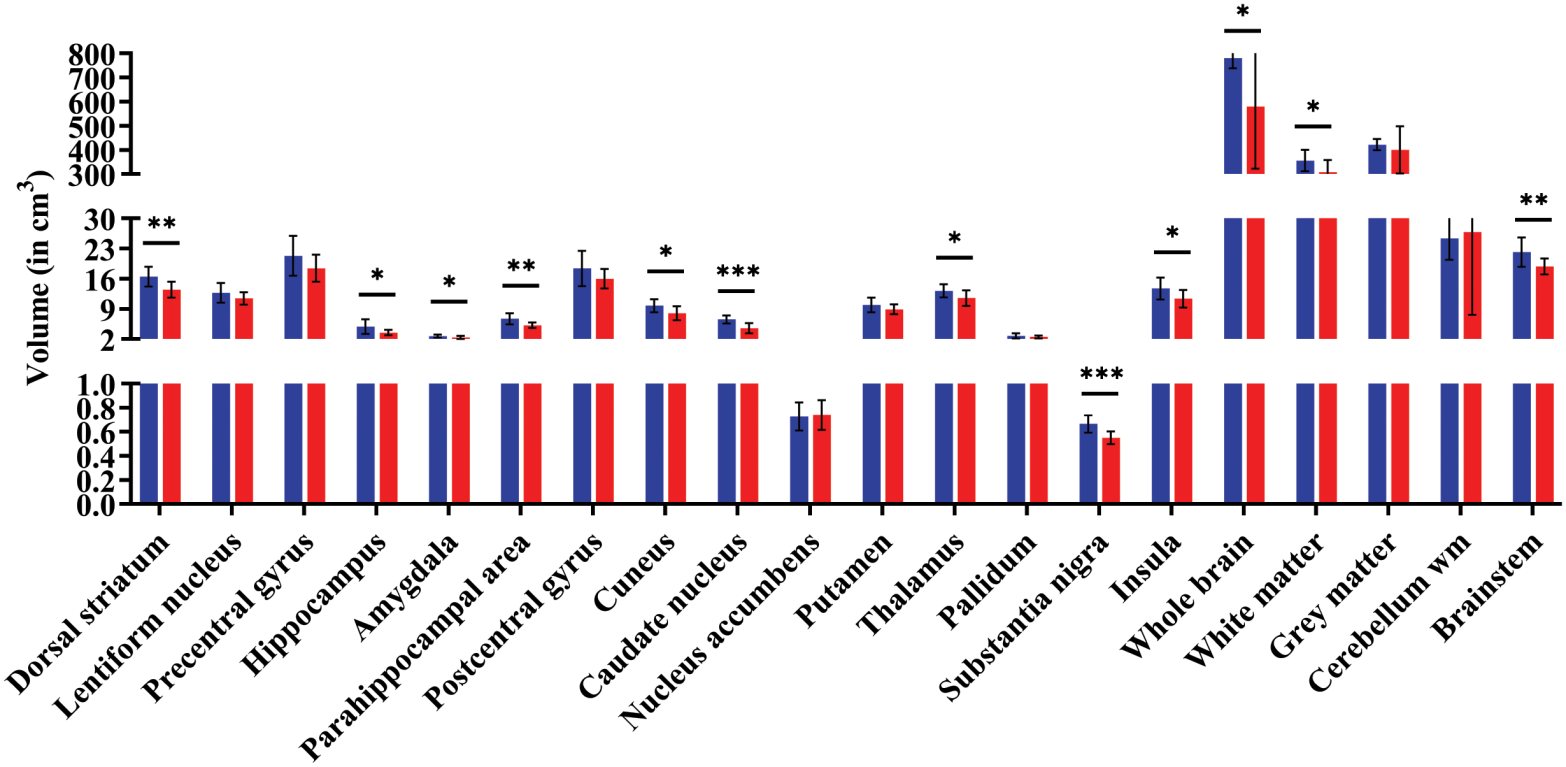
Comparisons of volumes (cm^3^) of twenty brain regions between PSP-S and disease controls. Significance level *<0.05; **<0.01; ***<0.001.

Volumetric measurements were obtained for 20 brain regions corresponding to the PET-assessed regions in PSP-S patients and disease controls. Notably, patients with PSP-S showed significant volumetric reductions in multiple brain regions, including dorsal striatum (*p* = 0.002), hippocampus (*p* = 0.023), amygdala (*p* = 0.032), parahippocampal area (*p* = 0.002), cuneus (*p* = 0.017), caudate nucleus (*p* < 0.001), thalamus (*p* = 0.024), substantia nigra (*p* < 0.001), insula (*p* = 0.022), WM (*p* = 0.025), brainstem (*p* = 0.008), and whole brain (*p* = 0.022) compared to disease controls (**Figure 5** and **Supplementary Table 18**). Among these, all the regions except amygdala remained significant after FDR correction. These regions were further correlated with significantly altered plasma and CSF cytokines as well as the clinical profile of the patients (**Supplementary Table 19**). Interestingly, we observed that the plasma IL-6 levels were inversely associated with the volumes of the lentiform nucleus (rho=-0.646, *p* = 0.023), nucleus accumbens (rho=-0.656, *p* = 0.021), putamen (rho=-0.646, *p* = 0.023), and pallidum (rho=-0.662, *p* = 0.019) (**Supplementary Figures 3A–D**). Plasma levels of IL-1β and IL-33 positively correlated with the grey matter (rho=0.653, *p* = 0.021) and caudate nucleus (rho=0.829, *p* = 0.021) volumes, respectively (**Supplementary Figure 3E** and **Supplementary Table 19**). However, no such correlations were observed for the cytokine levels in the CSF. In addition, we identified significant correlations between brain volumes and clinical parameters, including a positive correlation between WM and MoCA score (rho=0.618, *p* = 0.032); a negative correlation between grey matter and brainstem volumes with PSPRS component 3 (rho=-0.623, *p* = 0.031), and component 4 (rho=-0.591, *p* = 0.043), respectively, in PSP-S patients (**Supplementary Figures 3F–H** and **Supplementary Table 20**). However, none of these remained significant after FDR correction.

## 4 Discussion

Important pathobiological implications of immune cells and inflammatory mediators are increasingly being recognized in tauopathies. The current study adopted a unique integrative approach to delineate the missing link between peripheral inflammation and neuroinflammation in PSP-S. Multiple novel findings emerged from this combined immunological and neuroimaging study. The most notable finding was the heightened systemic inflammatory state in patients with PSP-S due to activation of NF-κB, inflammasome, and Th17 pathways. The expressions of *Nfkb1*, three key components of inflammasome (IL-1β, IL-18, and Caspase-1), and inflammatory Th17 pathway-related genes (*Il1b* and *Il6*) were significantly upregulated in patients with PSP-S.

Gene expression reflects the cellular state, and gene expression analyses are widely used to elucidate the molecular mechanisms underlying human diseases. Differential expression of genes also provides a basis for understanding the biological differences between healthy and disease states, as well as genetic architecture of complex diseases. In an earlier study, variants at PSP risk loci, such as *MAPT* and *MOBP*, were suggested to confer disease risk by influencing gene expression (32). No previous studies have investigated the expression patterns of three inflammatory pathway (NF-kB, inflammasome, and Th17)-related genes in the peripheral blood of patients with PSP-S. However, post-mortem analysis has demonstrated a topographical relationship between affected brain regions and patterns of cytokine expression. Specifically, IL-1β transcript levels were reported to be higher in the substantia nigra of patients with PSP-S than in those with Alzheimer’s disease (AD), whereas higher expression was observed in the parietal cortex of patients with AD compared to PSP (12). Increased expression of several immune-related genes, such as MIP-1α, MCP-1, IL-8, RANTES, IL-1β and IFN-γ were demonstrated in PBMCs of patients with PD (33). The fold changes observed for the significantly upregulated genes in patients with PSP-S in the present study are within the range typically reported in immune function-related gene expression studies and are likely to make biologically meaningful contribution to the on-going inflammatory milieu underlying neurodegenerative diseases such as PSP-S.

The current study also demonstrated significantly elevated plasma levels of inducer (IL-1β and IL-6) and effector (IL-17A and IL-17F) cytokines of the inflammatory Th17 cells in patients with PSP-S. To our knowledge, this is the first report demonstrating alterations of the components of three key inflammatory (NF-κB, inflammasome, and Th17) pathways in PSP-S. A recent study from India involving a relatively small cohort of patients with atypical parkinsonism, including PSP-S, examined IL-2, IL-4, IL-6, IL-10, IL-17A, IL-21, IL-22, IL-23, TNF, IFN-γ, GM-CSF, NF-κB, RORγt, and TGF-β, and reported elevated levels of only RORγt, IL-6, TNF, IL-10 and IL-4 (34). In contrast, levels of IL-4, IL-10, and TNF-α were not significantly elevated in our PSP-S cohort, whereas increased IL-6 levels was the only common finding between the two studies. The reasons for these discrepancies remain unclear; however, small sample size and inclusion of four distinct atypical parkinsonism phenotypes in the previous study may have contributed to the observed differences in immune marker profiles. The pathobiological relevance of the above inflammatory pathways in PSP-S is supported by substantial evidence from other pathological conditions of the brain (24, 35, 36). More importantly, these pathways exhibit complex multidimensional functional interactions with tau protein. A study on experimental animal suggests a role of pathological tau in activating NF-κB, thereby promoting neuroinflammation (37). The same study further showed that microglial NF-κB signaling mediates tau spreading and toxicity in tauopathy. Likewise, pathological tau activates the NLRP3 inflammasome, while NLRP3 inflammasome activation, in turn, drives tau pathology (38). Pathological tau also activates other inflammatory cytokines such as IL-1β via inflammasome pathway (39). In addition, recent studies reported an increased number of cytotoxic T cells in the mid brain of post-mortem brain samples of PSP-S patients (40, 41). Experimental evidence from AD rat model has also demonstrated that Th17 cells contribute to neuroinflammation and neurodegeneration in AD (42). Collectively, the above findings underscore an important pathological role of NF-κB, inflammasome and Th17 pathways in neuroinflammation and neurodegeneration. The current study provides unique findings on altered functions of these inflammatory pathways in the peripheral blood of patients with PSP-S, thereby strengthening the link between systemic immune dysregulation and the underlying disease pathology. Another notable finding of the current study was significantly increased CSF levels of inflammatory cytokines, such as IL-1β, IL-6, IL-17A, IL-22, and IL-23, in patients with PSP-S. An earlier study reported higher IL-6 levels but lower IL-1β levels in the CSF of patients with PSP-S (16). In contrast, a more recent study found no significant differences in CSF levels of IL-1β and IL-6 in patients with PSP-S compared to healthy controls (43). The CSF-based findings of the current study demonstrate evidence of neuroinflammation in PSP-S. Furthermore, a significant positive correlation in IL-1β levels was observed between plasma and CSF in patients with PSP-S. IL-1β has pleotropic functions and is a potent regulator of inflammatory response. The maturation and secretion of IL-1β is dependent on inflammasome activation, mediated by inflammatory caspases (44). Consistent with this mechanism, the expression of *Casp1* gene was found to be significantly upregulated in patients with PSP-S in the current study. In addition, IL-1β is a well-established inducer of the Th17 pathway, which was also found to be activated in patients with PSP-S. Collectively, these findings highlight IL-1β as a key mediator that may bridge peripheral inflammation and neuroinflammation in PSP-S.

The PET-MRI analysis provided several notable findings that further support the presence of neuroinflammation in PSP-S. The CSF levels of IL-1β and IL-6 exhibited significant correlation with the microglial activation profile in PSP-S patients. Another particularly interesting finding was the negative correlation between plasma IL-25 levels and neuroinflammation in the precentral gyrus in patients with PSP-S. Although IL-25 is a member of IL-17 family, it is known to inhibit inflammatory Th1 and Th17 pathways and thus it is a potential anti-inflammatory cytokine. This observation suggests an impaired anti-inflammatory defense in PSP-S, which may contribute to peripheral Th17 pathway activation and subsequent neuroinflammation. The current study also highlights the impact of neuroinflammation on the onset of the disease as higher neuroinflammation was associated with an early age at onset of PSP-S.

Other salient findings of the current study include structural brain changes, particularly significant volumetric reductions in various brain regions and their significant associations with inflammatory markers in PSP-S. Higher plasma IL-6 levels were associated with reduced volumes of the lentiform nucleus, nucleus accumbens, putamen, and pallidum in PSP-S patients. In a previous study, serum IL-6 concentration was positively correlated with the frontal horn width and negatively correlated with superior cerebellar area (23). Collectively, these findings suggest that inflammatory cytokines, such as IL-6, are associated with neurodegenerative changes in PSP-S. Furthermore, significant correlations were observed between WM volume and MoCA score, indicating that structural brain changes are linked to cognitive impairment. Overall, these findings suggest significant impact of inflammatory markers on the brain morphometric alterations, which, in turn, contribute to the core clinical manifestations of PSP-S.

## 5 Limitations

The present study demonstrates altered levels of three crucial determinants, such as NF-κB, inflammasome and Th17 pathway of inflammation and also suggests a possible impact of peripheral inflammatory markers on neuroinflammation in PSP-S. These are the novel findings and strengths of the current study. Notably, a recent study has demonstrated the importance of neuroimmune proteins in differentiating various tauopathies (45). Considering this and other developments in the field, the immune molecules seem to have immense value not only in understanding the underlying pathology but also in differential diagnosis of tauopathies. The current study also suggests the possibility of using the immune molecules of these pathways in biomarker discovery of PSP-S. However, the current proof-of-concept study linking peripheral inflammation to neuroinflammation as well as biomarker potential of these immune molecules in PSP-S needs further validation.

The current study has a few limitations. One of the major limitations is the heterogeneous nature of the disease control group, comprising PD and MSA. These disorders possess distinct inflammatory signatures as well as neurodegenerative mechanisms. Pooling them into a single disease control group might introduce confounding effects on the interpretation of the PET findings. The neuroimaging findings, although provided important insights into neuroinflammation, were conducted on a relatively small subset of PSP-S cases and compared with a heterogeneous disease control group, which may limit the generalizability of these observations. Therefore, the neuroimaging findings, their interpretation, and the conclusions drawn should be considered preliminary and interpreted with caution. The disease duration and severity vary significantly in the subtypes of PSP-S and these may substantially influence inflammatory molecules. Since the cohort size was relatively small, multivariable analysis controlling for age, sex, disease duration, and PSPRS score could not be performed. This limits the study from making more precise and stratified conclusion on the impact of inflammatory markers on disease subtypes and severity. Additionally, although the observed immune alterations appear promising as candidate biomarkers of PSP-S, their utility for disease diagnosis and monitoring of disease progression requires validation in larger, independent cohorts.

## Data Availability

The raw data supporting the conclusions of this article will be made available by the authors, without undue reservation.

## Supplementary material

Supplementary Figure 1 Linear correlation between plasma and CSF level of IL-1β.

Supplementary Figure 2 Correlation between SUV-R of various brain regions with **(B)** duration of the disease, **(A, C–F)** plasma level of cytokines, and **(G–J)** CSF level of cytokines. Significance level *p<0.05; **p<0.01; ***p<0.001.

Supplementary Figure 3 Correlation between volumes of various brain regions with **(A–E)** plasma level of cytokines, and **(F–H)** clinical variables. Significance level *p<0.05; **p<0.01; ***p<0.001.
