# Supplementary material for: Peripheral and central inflammation in progressive supranuclear palsy syndrome: an immunological and imaging neuroinflammation study
Source: Front Immunol. 2026 Jul 23;17:1841265. doi: 10.3389/fimmu.2026.1841265 (PMC13441997; doi:10.3389/fimmu.2026.1841265)
Supplement: Supplementary file 1 [file DataSheet1.pdf]

Supplementary Table 1: TaqMan assay IDs of the target genes and endogenous control.

SL no	Name of Gene	TaqMan Assay ID	Amplicon size (bp)	Design
1.	*Tbp**	Hs00427620_m1	91	Exon-Exon span
2.	*Tgfb*	Hs00998133_m1	57	Exon-Exon span
3.	*Il6*	Hs00985639_m1	66	Exon-Exon span
4.	*Stat3*	Hs00374280_m1	70	Exon-Exon span
5.	*Rorc*	Hs01076112_m1	62	Exon-Exon span
6.	*Il17*	Hs00174383_m1	80	Exon-Exon span
7.	*Il1b*	Hs01555410_m1	91	Exon-Exon span
8.	*Tnfa*	Hs00174128_m1	80	Exon-Exon span
9.	*Il22*	Hs01574154_m1	66	Exon-Exon span
10.	*Il23a*	Hs00372324_m1	107	Exon-Exon span
11.	*Il10*	Hs00961622_m1	74	Exon-Exon span
12.	*Nlrp3*	Hs00918082_m1	84	Exon-Exon span
13.	*Casp1*	Hs00354836_m1	76	Exon-Exon span
14.	*Il18*	Hs01038788_m1	115	Exon-Exon span
15.	*Nfkb1*	Hs00765730_m1	66	Exon-Exon span
16.	*Nfkb2*	Hs01028890_g1	91	Exon-Exon span

*- Endogenous control

Supplementary Table 2: Altered gene expression levels of the elements of the Th17-inflammasome axis between patients and the control group.

**Genes**	**Patients (n=56) Median (Q3, Q1)**	**Controls (n=56) Median (Q3, Q1)**	**Statistics**†	***p* value**	***Adj. p* value**
Il1b	1.458 (5.588, 0.401)	0.264 (5.72, 0.053)	-2.773	0.006**	0.045*
Il6	1.207 (3.4, 0.159)	0.242 (0.773, 0.105)	-2.159	0.031*	0.131
Il17	0.519 (2.227, 0.206)	0.345 (3.815, 0.134)	-0.642	0.521	0.703
Rorc	0.653 (1.515, 0.46)	0.981 (2.063, 0.573)	-1.418	0.156	0.347
Stat3	1.791 (3.33, 0.985)	2.044 (3.24, 1.289)	-0.695	0.487	0.703
Tgfb	3.52 (8.122, 1.805)	3.531 (10.196, 2.006)	-0.138	0.890	0.890
Tnfa	0.573 (1.719, 0.04)	0.272 (2.321, 0.136)	-0.580	0.562	0.703
Il22	1.593 (7.19, 0.502)	0.631 (4.174, 0.027)	-1.400	0.162	0.347
Casp1	15.719 (34.305, 6.712)	8.124 (15.814, 4.648)	-2.111	0.035*	0.131
Nlrp3	1.378 (2.613, 0.945)	1.396 (3.807, 0.681)	-0.283	0.777	0.880
Il10	0.122 (1.143, 0.041)	0.204 (0.918, 0.029)	-0.226	0.821	0.880
Il18	0.928 (2.348, 0.385)	0.368 (0.844, 0.246)	-3.355	0.001**	0.015*
Il23a	0.189 (0.611, 0.094)	0.251 (0.753, 0.112)	-0.693	0.488	0.703
Nfkb1	2.039 (2.678, 0.83)	0.855 (2.257, 0.535)	-1.985	0.047*	0.141
Nfkb2	33.472 (405.042, 0.976)	110.311 (559.076, 6.755)	-1.167	0.243	0.456

†-Mann-Whitney U test; Significance level *<0.05; **<0.01; ***<0.001

Supplementary Table 3: Differences in gene expression levels of the elements of the Th17-inflammasome axis between the PSP-RS and PSP-nonRS groups.

**Genes**	**PSP-RS (n=29) Median (Q3, Q1)**	**PSP-nonRS (n=27) Median (Q3, Q1)**	**Statistics**[†]	***p* value**	***Adj. p* value**
Il1b	1.279 (3.274, 0.309)	3.976 (11.023, 0.603)	-1.925	0.056	0.580
Il6	1.069 (3.539, 0.131)	1.359 (3.400, 0.159)	-0.339	0.735	0.788
Il17	0.428 (1.149, 0.186)	0.698 (3.22, 0.206)	-0.985	0.325	0.656
Rorc	0.891 (1.801, 0.424)	0.633 (1.369, 0.469)	-0.649	0.516	0.734
Stat3	2.084 (3.534, 1.092)	1.583 (2.635, 0.932)	-0.935	0.350	0.656
Tgfb	3.959 (8.104, 1.415)	3.027 (8.175, 2.045)	-0.355	0.723	0.788
Tnfa	0.117 (1.982, 0.039)	1.120 (1.637, 0.045)	-0.616	0.538	0.734
Il22	1.596 (12.194, 0.502)	1.511 (2.452, 0.348)	-0.968	0.333	0.656
Casp1	13.305 (25.510, 4.808)	21.028 (54.602, 7.230)	-0.961	0.337	0.656
Nlrp3	1.203 (2.281, 0.829)	1.498 (2.899, 1.157)	-1.324	0.185	0.656
Il10	0.274 (1.553, 0.054)	0.101 (0.852, 0.033)	-0.830	0.406	0.677
Il18	0.812 (1.978, 0.333)	0.928 (2.497, 0.430)	-0.374	0.708	0.788
Il23a	0.261 (0.621, 0.140)	0.130 (0.625, 0.070)	-1.573	0.116	0.580
Nfkb1	1.824 (2.526, 0.413)	2.379 (3.435, 1.073)	-1.621	0.105	0.580
Nfkb2	78.807 (304.451, 1.374)	27.540 (604.764, 0.976)	-0.261	0.794	0.794

†-Mann-Whitney U test

Supplementary Table 4: Respective FDR-adjusted p-values corresponding to the correlogram in **Figure 1f** (in PSP-S group).

**Genes**	***Il1b***	***Il6***	***Tnfa***	***Casp1***	***Nlrp3***	***Il18***	***Il23a***	***Nfkb1***
Il1b								
Il6	0.067							
Stat3	0.679	0.579						
Tgfb	0.579	0.579						
Tnfa	0.007**	0.007**						
Casp1	0.996							
Nlrp3				0.067				
Il18				0.480				
Il23a	0.546					0.007**		
Nfkb1	0.067	0.579	0.864		0.579		0.579	
Nfkb2	0.085	0.734						0.085

-Spearman's rank correlation; Significance level *<0.05; **<0.01; ***<0.001

Supplementary Table 5: Respective FDR-adjusted p-values corresponding to the correlogram in **Figure 1g** (in healthy control group).

**Genes**	***Il1b***	***Il6***	***Tnfa***	***Casp1***	***Nlrp3***	***Il18***	***Il23a***	***Nfkb1***
Il1b								
Il6	0.007**							
Stat3	0.038*	0.038*						
Tgfb	0.044*	0.038*						
Tnfa	0.215	0.038*						
Casp1	0.540							
Nlrp3				0.007**				
Il18				0.770				
Il23a	0.613					0.020*		
Nfkb1	0.676	0.540	0.007**		0.215		0.770	
Nfkb2	0.770	0.616						0.215

-Spearman's rank correlation; Significance level *<0.05; **<0.01; ***<0.001

Supplementary Table 6: Differences in observed plasma levels of cytokines between the patients and control groups.

Cytokines	Patients (n=56) Median (Q3, Q1)	Controls (n=56) Median (Q3, Q1)	Statistics[†]	*p* value	*Adj. p* value
**IL-1β**	1.32 (2.1, 0.563)	0.93 (1.5, 0.77)	-2.073	0.038*	0.098
**IL-4**	14.605 (28.24, 8.718)	17.875 (32.968, 6.46)	-0.185	0.853	0.853
**IL-6**	7.900 (14.035, 4.770)	5.120 (12.010, 1.810)	-2.585	0.010*	0.075
**IL-10**	30.975 (50.4, 15.798)	36.56 (84.13, 7.84)	-0.458	0.647	0.693
**IL-17A**	4.340 (6.915, 1.135)	0.760 (6.103, 0.490)	-2.259	0.024*	0.090
**IL-17F**	36.21 (156.17, 4.97)	13.295 (54.95, 0.8)	-2.910	0.004**	0.060
**IL-21**	50.92 (236.96, 29.66)	178.36 (271.195, 127.17)	-1.654	0.098	0.210
**IL-22**	27.6 (61.88, 4.78)	56.085 (75.83, 8.585)	-1.424	0.154	0.289
**IL-23**	89.6 (176.475, 25.215)	115.745 (162.76, 63.76)	-0.812	0.417	0.481
**IL-25**	1.680 (4.050, 0.460)	2.73 (7.150, 1.330)	-2.065	0.039*	0.098
**IL-31**	123 (240.965, 71.21)	170.94 (649.72, 0.49)	-0.835	0.404	0.481
**IL-33**	62.22 (97.61, 28.53)	99.255 (134.4, 60.865)	-2.430	0.015*	0.075
**IFN-γ**	22.09 (52.95, 6.97)	21.95 (44.25, 4.03)	-0.909	0.363	0.481
**sCD40L**	52.84 (148.935, 24.57)	106.11 (236.945, 21.468)	-0.943	0.346	0.481
**TNF-α**	19.585 (33.28, 14.21)	19.01 (31.483, 11.688)	-1.208	0.227	0.378

†-Mann-Whitney U test; Significance level *<0.05; **<0.01; ***<0.001

Supplementary Table 7: Differences in observed plasma levels of cytokines between the PSP-RS and PSP-nonRS groups.

**Cytokines**	**PSP-RS (n=29) Median (Q3, Q1)**	**PSP-nonRS (n=27) Median (Q3, Q1)**	**Statistics†**	***p* value**	***Adj. p* value**
**IL-1β**	1.260 (0.560, 2.100)	1.450 (0.570, 2.100)	-0.248	0.804	0.948
**IL-4**	15.605 (7.975, 30.653)	13.585 (10.485, 29.530)	-0.342	0.732	0.948
**IL-6**	7.510 (4.770, 17.270)	8.220 (5.680, 14.590)	-0.320	0.749	0.948
**IL-10**	30.450 (12.100, 40.120)	31.500 (17.170, 50.690)	-0.558	0.577	0.948
**IL-17A**	4.340 (0.605, 7.380)	5.120 (2.450, 7.380)	-0.354	0.723	0.948
**IL-17F**	63.130 (25.958, 119.500)	29.520 (0.800, 191.470)	-0.670	0.503	0.948
**IL-21**	44.740 (25.970, 257.595)	107.130 (26.090, 254.900)	-0.411	0.681	0.948
**IL-22**	28.430 (2.645, 61.605)	26.770 (15.900, 63.370)	-0.642	0.521	0.948
**IL-23**	102.170 (5.740, 130.300)	83.110 (63.760, 230.950)	-0.660	0.509	0.948
**IL-25**	2.240 (0.460, 4.150)	1.260 (0.460, 4.120)	-0.135	0.893	0.948
**IL-31**	170.940 (78.315, 256.085)	103.300 (71.210, 185.650)	-1.208	0.227	0.948
**IL-33**	47.020 (12.700, 104.430)	66.765 (28.530, 85.120)	-0.164	0.869	0.948
**IFN-γ**	14.710 (6.970, 53.170)	22.230 (7.600, 62.375)	-0.317	0.751	0.948
**sCD40L**	48.405 (16.580, 144.330)	52.840 (29.440, 158.240)	-0.506	0.613	0.948
**TNF-α**	18.830 (13.595, 52.730)	20.340 (15.770, 31.530)	-0.066	0.948	0.948

†-Mann-Whitney U test

Supplementary Table 8: Respective FDR adjusted p-values corresponding to the correlogram in **Figure 1g** (in PSP-S group).

**Cytokines**	**IL-1β**	**IL-4**	**IL-6**	**IL-10**	**IL-17A**	**IL-17F**	**IL-21**	**IL-22**	**IL-23**
**IL-1β**									
**IL-4**									
**IL-6**	0.987								
**IL-10**	0.821		0.516						
**IL-17A**	0.453	0.959	0.004**						
**IL-17F**	0.407	0.052	0.004**						
**IL-21**			0.038*		0.987	0.022*			
**IL-22**	0.004**		0.987		0.617				
**IL-23**	0.959				0.073	0.004**			
**IL-25**		0.062							
**IL-33**		0.868							
**TNF-α**	0.062		0.004**		0.004**	0.004**			0.022*

-Spearman's rank correlation; Significance level *<0.05; **<0.01; ***<0.001

Supplementary Table 9: Respective FDR adjusted p-values corresponding to the correlogram in **Figure 1g** (in healthy control group).

**Cytokines**	**IL-1β**	**IL-4**	**IL-6**	**IL-10**	**IL-17A**	**IL-17F**	**IL-21**	**IL-22**	**IL-23**
**IL-1β**									
**IL-4**									
**IL-6**	0.405								
**IL-10**	0.595		0.192						
**IL-17A**	0.384	0.694	0.004**						
**IL-17F**	0.595	0.004**	0.004**						
**IL-21**			0.100		0.423	0.163			
**IL-22**	0.127		0.100		0.004**				
**IL-23**	0.260				0.006**	0.011*			
**IL-25**		0.040*							
**IL-33**		0.324							
**TNF-α**	0.405		0.004**		0.004**	0.004**			0.043*

-Spearman's rank correlation; Significance level *<0.05; **<0.01; ***<0.001

Supplementary Table 10: Linear correlation of plasma level of IL-1β, IL-6, IL-17A, and IL-17F with their respective gene expression level in patients and healthy control groups.

**Groups**		**IL-1β**	**IL-6**	**IL-17A**	**IL-17F**
**Patients**	**rho**	0.066	-0.297	0.313	0.010
	***p*-value**	0.670	0.059	0.059	0.964
**Healthy controls**	**rho**	-0.137	-0.035	0.130	-0.013
	***p*-value**	0.391	0.835	0.632	0.962

-Spearman's rank correlation

Supplementary Table 11: Linear correlation between plasma and CSF level of cytokines in PSP-S.

Cytokines	rho	*p*-value	*Adj. p* value
**IL-1β**	0.602	0.004**	0.060
**IL-4**	0.676	0.140	0.429
**IL-6**	0.293	0.138	0.429
**IL-10**	-0.529	0.143	0.429
**IL-17A**	0.089	0.674	0.756
**IL-17F**	0.800	0.104	0.429
**IL-21**	0.393	0.185	0.456
**IL-22**	0.355	0.213	0.456
**IL-23**	0.102	0.739	0.756
**IL-25**	-0.081	0.756	0.756
**IL-31**	-0.313	0.298	0.559
**IL-33**	0.285	0.535	0.756
**IFN-γ**	0.242	0.426	0.710
**sCD40L**	0.214	0.610	0.756
**TNF-α**	-0.105	0.641	0.756

-Spearman's rank correlation; Significance level *<0.05; **<0.01; ***<0.001

Supplementary Table 12: Correlation between plasma cytokine levels and clinical variables in patients with PSP-S.

**Clinical variables**		**IL-1β**	**IL-6**	**IL-17A**	**IL-17F**	**IL-25**	**IL-33**
**Age of onset (year)**	rho	0.121	-0.095	-0.077	-0.047	0.068	-0.253
	p-value	0.373	0.492	0.583	0.732	0.650	0.156
**Duration of disease (months)**	rho	0.093	0.125	-0.050	-0.055	-0.054	-0.014
	p-value	0.497	0.367	0.725	0.691	0.720	0.936
**MoCA**	rho	-0.056	0.038	-0.035	0.083	-0.052	-0.005
	p-value	0.681	0.786	0.803	0.547	0.728	0.979
**PSPRS**	rho	0.508***	0.199	-0.017	0.249	0.034	-0.270
	p-value	<0.001	0.150	0.906	0.067	0.820	0.129
**PSPRS_C1**	rho	0.308*	-0.077	-0.128	0.061	-0.119	-0.450**
	p-value	0.021	0.578	0.363	0.656	0.425	0.009
**PSPRS_C2**	rho	0.109	0.197	0.079	0.207	0.099	0.091
	p-value	0.422	0.153	0.574	0.130	0.507	0.614
**PSPRS_C3**	rho	0.307*	0.083	-0.033	-0.017	-0.143	-0.129
	p-value	0.021	0.550	0.817	0.902	0.338	0.474
**PSPRS_C4**	rho	0.403**	0.353**	0.159	0.324*	0.167	-0.181
	p-value	0.002	0.009	0.255	0.016	0.263	0.315
**PSPRS_C5**	rho	0.333*	0.192	0.129	0.194	0.134	0.006
	p-value	0.012	0.164	0.357	0.157	0.371	0.976
**PSPRS_C6**	rho	0.490***	0.056	-0.152	0.154	-0.029	-0.330
	p-value	<0.001	0.690	0.278	0.260	0.849	0.061
**UPDRS-III**	rho	0.361**	0.157	0.002	0.165	0.138	-0.278
	p-value	0.006	0.256	0.989	0.230	0.355	0.117

-Spearman's rank correlation; Significance level *<0.05; **<0.01; ***<0.001

Supplementary Table 13: Correlation between CSF cytokine levels and clinical variables in patients with PSP-S.

**Clinical variables**		**IL-1β**	**IL-6**	**IL-17A**	**IL-17F**	**IL-25**	**IL-33**
**Age of onset (year)**	rho	0.335	-0.170	-0.001	-0.500	-0.055	-0.059
	p-value	0.138	0.386	0.997	0.450	0.822	0.881
**Duration of disease (months)**	rho	-0.101	-0.154	-0.274	0.200	0.342	0.164
	p-value	0.662	0.433	0.159	0.783	0.152	0.674
**MoCA**	rho	0.380	0.127	0.047	0.300	-0.379	-0.017
	p-value	0.089	0.520	0.811	0.683	0.109	0.966
**PSPRS**	rho	0.471*	0.487**	0.127	-0.100	0.195	0.201
	p-value	0.031	0.009	0.520	0.950	0.425	0.604
**PSPRS_C1**	rho	0.202	0.080	0.161	-0.264	0.190	-0.120
	p-value	0.381	0.684	0.414	0.668	0.435	0.759
**PSPRS_C2**	rho	0.227	0.228	0.213	-0.051	0.412	0.457
	p-value	0.322	0.244	0.277	0.935	0.079	0.216
**PSPRS_C3**	rho	0.141	0.040	0.296	0.564	0.129	-0.085
	p-value	0.541	0.840	0.126	0.322	0.600	0.827
**PSPRS_C4**	rho	0.507*	0.324	-0.023	0.308	0.235	0.272
	p-value	0.019	0.092	0.906	0.614	0.332	0.478
**PSPRS_C5**	rho	0.280	0.514**	0.409*	0.100	-0.049	0.295
	p-value	0.218	0.005	0.031	0.950	0.842	0.441
**PSPRS_C6**	rho	0.564**	0.719***	-0.046	-0.359	0.169	0.212
	p-value	0.008	<0.001	0.817	0.553	0.490	0.584
**UPDRS-III**	rho	0.457*	0.554**	0.342	-0.200	-0.032	0.609
	p-value	0.037	0.002	0.075	0.783	0.896	0.082

-Spearman's rank correlation; Significance level *<0.05; **<0.01; ***<0.001

Supplementary Table 14: Correlation between gene expression levels and clinical variables in patients with PSP-S.

**Clinical variables**		***Il1b***	***Il6***	***Casp1***	***Il18***	***Nfkb1***
**Age of onset (year)**	rho	0.005	0.030	0.181	0.065	0.164
	p-value	0.973	0.851	0.204	0.641	0.361
**Duration of disease (months)**	rho	0.098	-0.152	-0.133	-0.144	-0.005
	p-value	0.526	0.343	0.352	0.304	0.979
**MoCA**	rho	0.063	0.045	0.133	-0.184	0.095
	p-value	0.682	0.781	0.351	0.186	0.599
**PSPRS**	rho	-0.083	0.114	0.082	0.131	-0.002
	p-value	0.593	0.478	0.566	0.351	0.991
**PSPRS_C1**	rho	-0.194	0.083	0.070	0.242	-0.260
	p-value	0.206	0.604	0.626	0.081	0.144
**PSPRS_C2**	rho	-0.161	0.098	0.049	0.053	-0.122
	p-value	0.298	0.544	0.732	0.708	0.499
**PSPRS_C3**	rho	-0.074	0.065	0.087	-0.054	-0.108
	p-value	0.631	0.685	0.543	0.701	0.548
**PSPRS_C4**	rho	-0.028	-0.051	0.115	-0.034	0.148
	p-value	0.859	0.750	0.423	0.808	0.412
**PSPRS_C5**	rho	-0.177	0.230	0.180	0.180	0.131
	p-value	0.251	0.147	0.206	0.196	0.468
**PSPRS_C6**	rho	0.029	0.148	0.006	0.078	0.048
	p-value	0.853	0.357	0.968	0.579	0.792
**UPDRS-III**	rho	-0.084	0.328*	-0.020	0.038	0.117
	p-value	0.589	0.037	0.889	0.786	0.518

-Spearman's rank correlation; Significance level *<0.05; **<0.01; ***<0.001

Supplementary Table 15: Differences in SUV-R in twenty brain regions between the patients with PSP-S and diseased controls.

Brain regions	Patients (n=12)	Controls (n=11)	Statistics†	*p* value	*Adj. p* value
**Dorsal striatum**	0.814±0.058	0.837±0.052	-0.951	0.824	0.999
**Lentiform nucleus**	0.889±0.058	0.898±0.044	-0.379	0.646	0.999
**Precentral gyrus**	0.824±0.037	0.845±0.058	-1.022	0.841	0.999
**Hippocampus**	0.821±0.055	0.843±0.089	-0.723	0.761	0.999
**Amygdala**	0.805±0.065	0.827±0.073	-0.744	0.767	0.999
**Parahippocampal area**	0.800±0.077	0.795±0.047	0.206	0.419	0.999
**Postcentral gyrus**	0.807±0.027	0.827±0.065	-0.991	0.833	0.999
**Cuneus**	0.912±0.031	0.916±0.063	-0.194	0.576	0.999
**Caudate nucleus**	0.633±0.093	0.716±0.074	-2.336	0.985	0.999
**Nucleus accumbens**	0.839±0.067	0.943±0.099	-2.891	0.995	0.999
**Putamen**	0.900±0.055	0.926±0.042	-1.259	0.889	0.999
**Thalamus**	0.892±0.062	0.985±0.057	-3.751	0.999	0.999
**Pallidum**	0.850±0.075	0.802±0.085	1.432	0.083	0.553
**Substantia nigra**	0.848±0.122	0.830±0.063	0.403	0.346	0.999
**Insula**	0.795±0.065	0.847±0.066	-1.907	0.965	0.999
**Whole brain**	0.778±0.038	0.764±0.043	0.787	0.221	0.999
**White matter (wm)**	0.711±0.058	0.659±0.052	2.232	0.018*	0.180
**Grey matter**	0.835±0.027	0.839±0.062	-0.223	0.587	0.999
**Cerebellum wm**	0.898±0.072	0.836±0.054	2.345	0.014*	0.180
**Brainstem**	0.897±0.057	0.931±0.067	-1.296	0.896	0.999

†-Independent sample t-test (one-tailed); Significance level *<0.05; **<0.01; ***<0.001

Supplementary Table 16: Correlation between plasma and CSF cytokine levels and SUV-R in twenty brain regions in patients with PSP-S.

**Regions**		**Plasma**						**CSF**	
		**IL-1β**	**IL-6**	**IL-17A**	**IL-17F**	**IL-25**	**IL-33**	**IL-1β**	**IL-6**
**Dorsal striatum**	rho	0.135	0.335	0.175	0.080	0.124	0.342	0.000	0.700
	p-value	0.337	0.143	0.293	0.403	0.642	0.774	1.000	0.188
**Lentiform nucleus**	rho	0.200	0.226	-0.018	-0.102	0.041	-0.162	0.500	0.300
	p-value	0.267	0.240	0.522	0.623	0.548	0.364	0.391	0.624
**Precentral gyrus**	rho	-0.064	-0.346	-0.514	-0.537	-0.740**	-0.126	0.100	0.100
	p-value	0.579	0.864	0.956	0.964	0.005	0.394	0.873	0.873
**Hippocampus**	rho	0.228	0.106	0.254	0.203	0.041	-0.198	0.100	0.400
	p-value	0.238	0.372	0.213	0.263	0.548	0.335	0.873	0.505
**Amygdala**	rho	0.300	0.085	0.325	0.160	0.078	0.000	0.000	0.700
	p-value	0.172	0.397	0.151	0.310	0.590	0.500	1.000	0.188
**Parahippocampal area**	rho	-0.011	0.247	0.218	0.239	0.189	0.108	-0.200	0.700
	p-value	0.513	0.220	0.248	0.227	0.711	0.591	0.747	0.188
**Postcentral gyrus**	rho	0.111	-0.176	-0.089	-0.203	-0.529*	-0.018	0.700	0.000
	p-value	0.366	0.708	0.609	0.737	0.047	0.485	0.188	1.000
**Cuneus**	rho	0.036	-0.670	-0.664	-0.457	-0.510	-0.559	0.200	0.300
	p-value	0.456	0.991	0.991	0.932	0.054	0.096	0.747	0.624
**Caudate nucleus**	rho	-0.021	0.473	0.282	0.109	0.193	0.559	-0.100	0.600
	p-value	0.526	0.060	0.187	0.368	0.715	0.904	0.873	0.285
**Nucleus accumbens**	rho	0.396	-0.243	-0.307	-0.616	-0.625*	-0.162	1.000*	-0.300
	p-value	0.101	0.777	0.834	0.984	0.020	0.364	0.017	0.624
**Putamen**	rho	0.171	0.212	-0.036	-0.131	0.041	-0.216	0.500	0.300
	p-value	0.297	0.255	0.544	0.657	0.548	0.321	0.391	0.624
**Thalamus**	rho	0.339	-0.032	-0.386	-0.551	-0.460	-0.450	0.800	-0.300
	p-value	0.141	0.539	0.892	0.968	0.077	0.155	0.104	0.624
**Pallidum**	rho	0.371	-0.152	-0.143	-0.254	-0.207	-0.703*	0.700	0.300
	p-value	0.118	0.681	0.671	0.787	0.271	0.039	0.188	0.624
**Substantia nigra**	rho	-0.189	0.437	0.250	0.261	0.294	0.270	0.100	0.400
	p-value	0.722	0.078	0.217	0.206	0.810	0.721	0.873	0.505
**Insula**	rho	0.157	0.310	0.336	0.261	0.326	0.180	0.000	0.700
	p-value	0.313	0.163	0.143	0.206	0.836	0.650	1.000	0.188
**Whole brain**	rho	0.300	-0.018	0.279	0.080	0.018	-0.342	-0.300	1.000*
	p-value	0.172	0.522	0.190	0.403	0.521	0.226	0.624	0.017
**White matter (wm)**	rho	0.232	0.145	0.482	0.384	0.230	-0.144	-0.300	1.000*
	p-value	0.234	0.327	0.056	0.109	0.752	0.379	0.624	0.017
**Grey matter**	rho	0.314	-0.317	-0.289	-0.624	-0.662*	-0.595	0.700	0.300
	p-value	0.160	0.843	0.819	0.985	0.013	0.080	0.188	0.624
**Cerebellum wm**	rho	0.148	0.164	0.496	0.407	0.295	-0.342	-0.600	0.900*
	p-value	0.323	0.305	0.051	0.095	0.811	0.226	0.285	0.037
**Brainstem**	rho	0.057	0.473	0.197	0.186	0.337	0.162	-0.200	0.700
	p-value	0.430	0.060	0.270	0.282	0.845	0.636	0.747	0.188

-Spearman's rank correlation; Significance level *<0.05; **<0.01; ***<0.001

Supplementary Table 17: Correlation between clinical variables and SUV-R in twenty brain regions in patients with PSP-S.

Brain regions		Age of onset (year)	Duration of disease	MoCA	PSPRS	PSPRS_C1	PSPRS_C2	PSPRS_C3	PSPRS_C4	PSPRS_C5	PSPRS_C6	UPDRS - III
**Dorsal striatum**	**rho**	-0.403	0.125	0.067	0.021	0.056	0.098	-0.026	0.050	0.280	0.028	0.039
	p	0.194	0.699	0.837	0.948	0.862	0.762	0.935	0.878	0.378	0.931	0.905
**Lentiform nucleus**	**rho**	-0.490	0.061	0.011	-0.268	-0.127	-0.256	0.183	-0.135	0.122	-0.254	-0.179
	p	0.106	0.851	0.974	0.400	0.695	0.421	0.570	0.677	0.705	0.425	0.579
**Precentral gyrus**	**rho**	-0.165	0.604*	-0.151	-0.085	-0.053	-0.173	-0.284	0.188	-0.327	0.032	-0.235
	p	0.609	0.038	0.640	0.794	0.870	0.590	0.371	0.559	0.300	0.922	0.463
**Hippocampus**	**rho**	-0.792**	0.221	0.182	0.338	0.440	-0.068	0.358	0.205	0.133	0.311	0.049
	p	0.002	0.489	0.570	0.283	0.152	0.834	0.253	0.522	0.681	0.325	0.880
**Amygdala**	**rho**	-0.697*	0.304	0.340	0.261	0.440	0.038	0.235	-0.071	0.341	0.307	0.273
	p	0.012	0.337	0.279	0.413	0.152	0.907	0.462	0.827	0.278	0.331	0.390
**Parahippocampal area**	**rho**	-0.736**	0.296	0.228	0.324	0.493	0.181	0.151	0.064	0.352	0.258	0.347
	p	0.006	0.349	0.476	0.304	0.103	0.574	0.640	0.844	0.262	0.418	0.269
**Postcentral gyrus**	**rho**	0.035	0.343	-0.316	0.148	-0.123	-0.136	0.239	0.503	-0.223	0.184	-0.329
	p	0.914	0.275	0.317	0.646	0.703	0.674	0.455	0.096	0.487	0.568	0.296
**Cuneus**	**rho**	0.014	-0.050	-0.161	-0.430	-0.440	-0.362	-0.407	-0.280	-0.478	-0.106	-0.305
	p	0.966	0.877	0.616	0.163	0.152	0.248	0.189	0.379	0.116	0.743	0.336
**Caudate nucleus**	**rho**	-0.340	0.100	0.323	0.148	-0.014	0.498	-0.516	0.195	0.467	-0.018	0.109
	p	0.280	0.757	0.306	0.646	0.965	0.100	0.086	0.544	0.126	0.957	0.737
**Nucleus accumbens**	**rho**	-0.399	0.518	0.425	-0.408	-0.254	-0.196	-0.288	-0.414	0.079	-0.124	-0.175
	p	0.198	0.085	0.169	0.187	0.427	0.541	0.364	0.181	0.807	0.702	0.586
**Putamen**	**rho**	-0.448	0.018	-0.018	-0.317	-0.173	-0.256	-0.302	-0.166	0.097	-0.332	-0.193
	p	0.144	0.956	0.957	0.316	0.592	0.421	0.340	0.605	0.764	0.291	0.549
**Thalamus**	**rho**	-0.364	0.347	0.228	-0.542	-0.426	-0.354	0.025	-0.273	0.036	-0.290	-0.368
	p	0.244	0.270	0.476	0.069	0.167	0.258	0.940	0.391	0.912	0.361	0.240
**Pallidum**	**rho**	-0.546	0.157	0.042	-0.317	-0.331	-0.362	-0.470	-0.173	0.097	-0.138	-0.231
	p	0.066	0.626	0.897	0.316	0.293	0.248	0.123	0.590	0.764	0.669	0.470
**Substantia nigra**	**rho**	-0.452	-0.032	-0.095	0.183	0.327	0.136	-0.147	0.212	0.158	-0.134	0.130
	p	0.140	0.921	0.770	0.569	0.299	0.674	0.648	0.507	0.624	0.677	0.688
**Insula**	**rho**	-0.588*	0.029	0.239	0.190	0.173	0.196	0.442	0.067	0.402	0.141	0.165
	p	0.044	0.930	0.455	0.554	0.592	0.541	0.150	0.835	0.195	0.661	0.609
**Whole brain**	**rho**	-0.564	0.354	0.025	0.120	0.320	-0.106	0.389	-0.092	0.338	0.173	0.319
	p	0.056	0.259	0.940	0.711	0.310	0.744	0.211	0.776	0.283	0.590	0.313
**White matter (wm)**	**rho**	-0.529	0.154	-0.130	0.352	0.440	-0.060	0.354	0.241	0.338	0.276	0.315
	p	0.077	0.634	0.688	0.262	0.152	0.852	0.258	0.451	0.283	0.386	0.318
**Grey matter**	**rho**	-0.424	0.539	0.102	-0.423	-0.180	-0.324	-0.123	-0.428	-0.018	-0.247	-0.126
	p	0.170	0.070	0.753	0.171	0.577	0.304	0.704	0.165	0.956	0.438	0.696
**Cerebellum wm**	**rho**	-0.263	0.048	-0.462	0.307	0.265	-0.159	-0.242	0.456	0.272	0.113	0.212
	p	0.409	0.881	0.130	0.332	0.406	0.622	0.448	0.137	0.393	0.726	0.508
**Brainstem**	**rho**	-0.446	0.097	-0.046	-0.007	0.057	-0.015	-0.007	0.172	0.395	-0.105	0.091
	p	0.146	0.765	0.888	0.983	0.861	0.963	0.983	0.592	0.204	0.746	0.778

-Spearman's rank correlation; Significance level *<0.05; **<0.01; ***<0.001

Supplementary Table 18: Volumetric differences (in CC) in twenty brain regions between the patients with PSP-S and diseased controls.

**Brain regions**	**Patients (n=12)**	**Controls (n=11)**	**Statistics**[†]	***p* value**	***Adj. p* value**
**Dorsal striatum**	13.484±1.856	16.530±2.271	3.535	0.002**	0.010*
**Lentiform nucleus**	11.461±1.465	12.741±2.264	1.623	0.119	0.149
**Precentral gyrus**	18.447±3.144	21.356±4.609	1.754	0.095	0.136
**Hippocampus**	3.523±0.630	4.921±1.680	2.597	0.023*	0.045*
**Amygdala**	2.388±0.374	2.736±0.346	2.304	0.032*	0.053
**Parahippocampal area**	5.247±0.656	6.729±1.321	3.453	0.002**	0.010*
**Postcentral gyrus**	16.017±2.249	18.412±4.089	1.762	0.093	0.136
**Cuneus**	8.021±1.639	9.781±1.505	2.600	0.017*	0.045*
**Caudate nucleus**	4.549±1.181	6.583±0.945	4.529	<0.001***	0.010*
**Nucleus accumbens**	0.740±0.124	0.728±0.117	-0.219	0.829	0.829
**Putamen**	8.934±1.129	9.947±1.695	1.699	0.104	0.139
**Thalamus**	11.536±1.781	13.215±1.497	2.434	0.024*	0.045*
**Pallidum**	2.527±0.343	2.794±0.575	1.366	0.186	0.219
**Substantia nigra**	0.550±0.053	0.665±0.073	4.164	<0.001***	0.010*
**Insula**	11.400±2.043	13.759±2.541	2.463	0.022*	0.045*
**Whole brain**	580.322±257.195	780.568±42.177	2.637	0.022*	0.045*
**White matter (wm)**	308.598±50.592	356.506±43.808	2.417	0.025*	0.045*
**Grey matter**	400.881±97.369	422.686±23.208	0.741	0.472	0.524
**Cerebellum wm**	26.836±19.171	25.350±4.936	-0.249	0.806	0.829
**Brainstem**	18.904±1.859	22.217±3.399	2.934	0.008**	0.032*

†-Independent sample t-test; Significance level *<0.05; **<0.01; ***<0.001

Supplementary Table 19: Correlation between plasma and CSF cytokine levels and volumes of twenty brain regions in patients with PSP-S.

**Brain volumes (in CM³)**		**Plasma**						**CSF**	
		**IL-1β**	**IL-6**	**IL-17A**	**IL-17F**	**IL-25**	**IL-33**	**IL-1β**	**IL-6**
**Dorsal striatum**	rho	0.139	-0.155	0.036	-0.073	-0.147	-0.090	-0.300	-0.800
	p-value	0.666	0.630	0.912	0.823	0.666	0.848	0.683	0.133
**Lentiform nucleus**	rho	0.439	-0.646*	-0.193	-0.326	-0.395	-0.613	0.100	-0.500
	p-value	0.154	0.023	0.548	0.301	0.229	0.144	0.950	0.450
**Precentral gyrus**	rho	0.278	-0.328	-0.100	-0.334	-0.336	0.324	-0.300	0.300
	p-value	0.381	0.298	0.757	0.289	0.313	0.478	0.683	0.683
**Hippocampus**	rho	0.300	-0.423	-0.018	-0.297	-0.253	-0.144	-0.600	-0.100
	p-value	0.344	0.170	0.956	0.348	0.453	0.758	0.350	0.950
**Amygdala**	rho	0.236	-0.488	-0.120	-0.276	-0.350	-0.055	-0.564	-0.205
	p-value	0.461	0.108	0.711	0.385	0.291	0.908	0.322	0.741
**Parahippocampal area**	rho	0.253	-0.469	-0.193	-0.464	-0.372	-0.252	-0.500	-0.300
	p-value	0.427	0.124	0.548	0.129	0.259	0.585	0.450	0.683
**Postcentral gyrus**	rho	0.246	-0.346	-0.139	-0.428	-0.336	0.270	-0.300	0.300
	p-value	0.441	0.271	0.666	0.165	0.313	0.558	0.683	0.683
**Cuneus**	rho	-0.132	-0.258	0.043	-0.160	-0.308	0.054	-0.800	0.300
	p-value	0.683	0.419	0.895	0.620	0.357	0.908	0.133	0.683
**Caudate nucleus**	rho	-0.171	0.148	0.050	0.000	0.009	0.829*	-0.500	-0.400
	p-value	0.595	0.646	0.877	1.000	0.979	0.021	0.450	0.517
**Nucleus accumbens**	rho	0.406	-0.656*	-0.171	-0.196	-0.405	-0.523	0.100	-0.500
	p-value	0.190	0.021	0.594	0.542	0.217	0.229	0.950	0.450
**Putamen**	rho	0.439	-0.646*	-0.193	-0.326	-0.395	-0.613	0.100	-0.500
	p-value	0.154	0.023	0.548	0.301	0.229	0.144	0.950	0.450
**Thalamus**	rho	0.168	-0.466	-0.132	-0.123	-0.221	-0.216	-0.200	-0.200
	p-value	0.603	0.127	0.682	0.703	0.514	0.641	0.783	0.783
**Pallidum**	rho	0.389	-0.663*	-0.236	-0.297	-0.382	-0.685	0.100	-0.500
	p-value	0.212	0.019	0.461	0.348	0.247	0.090	0.950	0.450
**Substantia nigra**	rho	0.103	-0.198	0.225	-0.087	-0.124	-0.018	-0.800	0.300
	p-value	0.749	0.538	0.482	0.788	0.716	0.969	0.133	0.683
**Insula**	rho	0.392	-0.533	-0.189	-0.421	-0.432	0.072	0.100	-0.100
	p-value	0.207	0.075	0.556	0.173	0.184	0.878	0.950	0.950
**Whole brain**	rho	0.111	-0.377	-0.179	-0.261	-0.230	-0.018	-0.300	-0.800
	p-value	0.732	0.226	0.579	0.412	0.496	0.969	0.683	0.133
**White matter (wm)**	rho	-0.089	-0.349	-0.414	-0.218	-0.326	0.108	0.300	-0.700
	p-value	0.783	0.266	0.180	0.497	0.327	0.818	0.683	0.233
**Grey matter**	rho	0.653*	-0.423	0.118	-0.138	-0.437	-0.126	0.300	0.200
	p-value	0.021	0.170	0.715	0.669	0.179	0.788	0.683	0.783
**Cerebellum wm**	rho	-0.185	-0.102	-0.121	-0.232	-0.009	0.144	-0.600	-0.500
	p-value	0.564	0.752	0.707	0.468	0.979	0.758	0.350	0.450
**Brainstem**	rho	0.004	-0.194	-0.154	-0.363	-0.156	0.018	-0.600	-0.500
	p-value	0.991	0.546	0.634	0.247	0.646	0.969	0.350	0.450

-Spearman's rank correlation; Significance level *<0.05; **<0.01; ***<0.001

Supplementary Table 20: Correlation between clinical variables and volumes of twenty brain regions in patients with PSP-S.

**Brain volumes**		**Age of onset (year)**	**Duration of disease**	**MoCA**	**PSPRS**	**PSPRS_C1**	**PSPRS_C2**	**PSPRS_C3**	**PSPRS_C4**	**PSPRS_C5**	**PSPRS_C6**	**UPDRS - III**
**Dorsal striatum**	**rho**	-0.011	0.061	0.404	0.092	0.144	0.023	0.166	-0.060	-0.054	0.124	-0.018
	p	0.974	0.851	0.193	0.777	0.654	0.944	0.607	0.853	0.868	0.702	0.957
**Lentiform nucleus**	**rho**	-0.172	0.129	0.379	-0.197	0.035	-0.392	-0.148	-0.496	-0.223	0.124	-0.063
	p	0.594	0.690	0.224	0.539	0.913	0.207	0.647	0.101	0.487	0.702	0.846
**Precentral gyrus**	**rho**	0.088	0.082	0.312	-0.232	0.085	-0.113	-0.202	-0.485	-0.201	-0.032	-0.014
	p	0.787	0.800	0.323	0.467	0.794	0.726	0.530	0.110	0.531	0.922	0.966
**Hippocampus**	**rho**	-0.077	0.046	0.432	-0.183	0.011	-0.060	-0.058	-0.453	-0.065	-0.071	0.042
	p	0.812	0.886	0.161	0.569	0.974	0.852	0.859	0.139	0.842	0.827	0.897
**Amygdala**	**rho**	-0.156	0.161	0.432	-0.049	0.131	-0.060	-0.038	-0.332	-0.139	0.119	0.051
	p	0.628	0.617	0.160	0.879	0.686	0.852	0.907	0.292	0.668	0.714	0.875
**Parahippocampal area**	**rho**	0.039	0.221	0.323	-0.331	-0.074	-0.151	0.007	-0.520	-0.072	-0.155	0.060
	p	0.905	0.489	0.306	0.293	0.819	0.640	0.982	0.083	0.824	0.629	0.854
**Postcentral gyrus**	**rho**	0.011	0.107	0.351	-0.317	0.011	-0.098	-0.227	-0.552	-0.172	-0.177	-0.021
	p	0.974	0.740	0.263	0.316	0.974	0.762	0.479	0.063	0.592	0.583	0.948
**Cuneus**	**rho**	-0.189	0.368	0.179	0.190	0.373	0.219	0.025	-0.067	0.040	0.021	0.343
	p	0.556	0.239	0.578	0.554	0.232	0.495	0.938	0.835	0.903	0.948	0.275
**Caudate nucleus**	**rho**	0.231	-0.029	0.316	0.127	0.116	0.332	0.407	0.096	0.004	-0.011	0.039
	p	0.470	0.930	0.317	0.695	0.719	0.292	0.190	0.768	0.991	0.974	0.905
**Nucleus accumbens**	**rho**	-0.193	0.214	0.232	0.021	0.208	-0.422	-0.169	-0.273	-0.291	0.375	-0.046
	p	0.549	0.504	0.469	0.948	0.517	0.172	0.599	0.391	0.359	0.230	0.888
**Putamen**	**rho**	-0.172	0.129	0.379	-0.197	0.035	-0.392	-0.148	-0.496	-0.223	0.124	-0.063
	p	0.594	0.690	0.224	0.539	0.913	0.207	0.647	0.101	0.487	0.702	0.846
**Thalamus**	**rho**	-0.109	0.261	0.340	0.021	0.289	-0.173	-0.022	-0.418	-0.022	0.269	0.277
	p	0.737	0.413	0.279	0.948	0.363	0.590	0.947	0.177	0.947	0.399	0.384
**Pallidum**	**rho**	-0.277	0.300	0.260	-0.134	0.148	-0.452	-0.173	-0.428	-0.169	0.208	0.056
	p	0.384	0.343	0.415	0.678	0.646	0.140	0.591	0.165	0.600	0.516	0.863
**Substantia nigra**	**rho**	-0.196	0.375	0.207	0.127	0.401	0.090	-0.184	-0.237	0.216	0.046	0.438
	p	0.541	0.230	0.519	0.695	0.196	0.780	0.568	0.458	0.501	0.887	0.155
**Insula**	**rho**	-0.018	-0.032	0.537	-0.232	-0.169	-0.045	-0.191	-0.453	-0.280	0.057	-0.235
	p	0.957	0.921	0.072	0.467	0.599	0.889	0.553	0.139	0.378	0.861	0.463
**Whole brain**	**rho**	-0.077	-0.064	0.407	-0.155	0.099	-0.106	0.065	-0.389	-0.284	-0.088	-0.070
	p	0.812	0.843	0.189	0.631	0.760	0.744	0.841	0.211	0.371	0.785	0.829
**White matter (wm)**	**rho**	-0.448	-0.143	0.618*	0.014	0.067	0.143	-0.108	-0.191	-0.413	0.117	-0.249
	p	0.144	0.658	0.032	0.965	0.836	0.657	0.738	0.552	0.182	0.718	0.436
**Grey matter**	**rho**	-0.042	0.161	0.165	0.085	0.025	-0.256	-0.623*	-0.120	-0.190	0.491	-0.231
	p	0.897	0.618	0.609	0.794	0.939	0.421	0.031	0.709	0.553	0.105	0.470
**Cerebellum wm**	**rho**	-0.140	0.296	0.396	-0.169	0.190	0.128	0.097	-0.513	0.255	-0.269	0.448
	p	0.664	0.349	0.202	0.599	0.554	0.691	0.764	0.088	0.424	0.399	0.144
**Brainstem**	**rho**	-0.175	0.268	0.425	-0.303	0.095	-0.008	-0.047	-0.591*	0.144	-0.322	0.280
	p	0.586	0.400	0.169	0.339	0.769	0.981	0.885	0.043	0.656	0.308	0.378

-Spearman's rank correlation; Significance level *<0.05; **<0.01; ***<0.001
